# Safety and efficacy of tofacitinib for up to 9.5 years in the treatment of rheumatoid arthritis: final results of a global, open-label, long-term extension study

**DOI:** 10.1186/s13075-019-1866-2

**Published:** 2019-04-05

**Authors:** Jürgen Wollenhaupt, Eun-Bong Lee, Jeffrey R. Curtis, Joel Silverfield, Ketti Terry, Koshika Soma, Chris Mojcik, Ryan DeMasi, Sander Strengholt, Kenneth Kwok, Irina Lazariciu, Lisy Wang, Stanley Cohen

**Affiliations:** 1 Rheumatologie Hamburg, Struenseehaus, Hamburg, Germany; 2 Seoul National University, Seoul, Republic of Korea; 3 University of Alabama at Birmingham, Birmingham, AL USA; 4 Healthpoint Medical Group, Tampa, FL USA; 5 Pfizer Inc, Groton, CT USA; 6 Pfizer Inc, New York, NY USA; 7 Pfizer Inc, Collegeville, PA USA; 8 Pfizer Inc, Capelle aan den Ijssel, Netherlands; 9 IQVIA Canada, Montréal, Quebec Canada; 10 Metroplex Clinical Research Center and University of Texas Southwestern Medical Center, Dallas, TX USA

**Keywords:** Rheumatoid arthritis, Tofacitinib, Long-term extension

## Abstract

**Background:**

Final data are presented for the ORAL Sequel long-term extension (LTE) study evaluating the safety and efficacy of tofacitinib 5 mg and 10 mg twice daily (BID) for up to 9.5 years in patients with rheumatoid arthritis (RA).

**Methods:**

Eligible patients had previously completed a phase 1, 2, or 3 qualifying index study of tofacitinib and received open-label tofacitinib 5 mg or 10 mg BID. Stable background therapy, including csDMARDs, was continued; adjustments to tofacitinib or background therapy were permitted at investigators’ discretion. Assignment to dose groups (5 mg or 10 mg BID) was based on patients’ average total daily dose. The primary objective was to determine the long-term safety and tolerability of tofacitinib 5 mg and 10 mg BID; the key secondary objective was to evaluate the long-term persistence of efficacy.

**Results:**

Between February 5, 2007, and November 30, 2016, 4481 patients were enrolled. Total tofacitinib exposure was 16,291 patient-years. Safety data are reported up to month 114 for all tofacitinib; efficacy data are reported up to month 96 for tofacitinib 5 mg BID and month 72 for 10 mg BID (with low patient numbers limiting interpretation beyond these time points). Overall, 52% of patients discontinued (24% due to adverse events [AEs] and 4% due to insufficient clinical response); the safety profile remained consistent with that observed in prior phase 1, 2, 3, or LTE studies. The incidence rate (IR; number of patients with events per 100 patient-years) for AEs leading to discontinuation was 6.8. For all-cause AEs of special interest, IRs were 3.4 for herpes zoster, 2.4 for serious infections, 0.8 for malignancies excluding non-melanoma skin cancer, 0.4 for major adverse cardiovascular events, and 0.3 for all-cause mortality. Clinically meaningful improvements in the signs and symptoms of RA and physical functioning, which were observed in the index studies, were maintained.

**Conclusions:**

Tofacitinib 5 mg and 10 mg BID demonstrated a consistent safety profile (as monotherapy or combination therapy) and sustained efficacy in this open-label LTE study of patients with RA. Safety data are reported up to 9.5 years, and efficacy data up to 8 years, based on adequate patient numbers to support conclusions.

**Trial registration:**

NCT00413699, funded by Pfizer Inc (date of trial registration: December 20, 2006)

**Electronic supplementary material:**

The online version of this article (10.1186/s13075-019-1866-2) contains supplementary material, which is available to authorized users.

## Background

Rheumatoid arthritis (RA) is a chronic and debilitating autoimmune disease characterized by systemic inflammation, persistent synovitis, and joint destruction, and affects an estimated 0.24% of the global population [1].

Tofacitinib is an oral Janus kinase inhibitor for the treatment of RA. The efficacy and safety of tofacitinib 5 mg and 10 mg twice daily (BID) administered as monotherapy or in combination with conventional synthetic disease-modifying antirheumatic drugs (csDMARDs), mainly methotrexate (MTX), in patients with moderately to severely active RA, have been demonstrated in phase 2 [2–6] and phase 3 [7–13] randomized controlled trials (RCTs) of up to 24 months’ duration.

The efficacy and safety of therapy is typically evaluated via double-blind RCTs. As RA is a chronic disease requiring long-term treatment, it is important to assess the long-term effectiveness and safety of RA therapies to understand the potential lifelong impact on patients’ health and quality of life. Of note, long-term extension (LTE) studies, with their controlled setting and rigorous safety reporting, offer the ability to observe and evaluate long-latency safety events, such as malignancies and cardiovascular events, as well as short-latency events, such as infections. Efficacy and safety data from LTE studies of tofacitinib treatment in global (Study A3921024; ORAL Sequel) and Japanese (Study A3921041) populations are part of the largest clinical development program undertaken for any RA treatment to date [14–16].

Interim results from the LTE studies have been regularly reported, and final results from Study A3921041, the LTE study conducted in Japanese patients, have been published [15]. Here, we report the final data from the global LTE ORAL Sequel study and describe the safety and efficacy of treatment with tofacitinib 5 mg and 10 mg BID for up to 9.5 years in patients with RA.

## Methods

### Study design and treatment

ORAL Sequel (NCT00413699; Study A3921024) was an open-label follow-up LTE study conducted in 414 centers across 43 countries (further details provided in Additional file 1: Table S1). Eligible patients had previously completed a prior qualifying index study of tofacitinib (Additional file 1: Table S1), which included two phase 1 studies, eight phase 2 studies, and six phase 3 studies.

Across qualifying index studies, tofacitinib was dosed at 1, 3, 5, 10, 15, and 30 mg BID, or 20 mg once daily, as monotherapy or in combination with background csDMARDs (mostly MTX).

The majority of enrolled patients from phase 2 qualifying index studies initiated open-label tofacitinib at 5 mg BID, and the majority of patients from phase 3 qualifying index studies initiated open-label tofacitinib at 10 mg BID, except for patients from China who initiated tofacitinib 5 mg BID as per the protocol. After LTE study baseline, the tofacitinib dose could be increased or decreased at the discretion of the investigator (e.g., increased in the case of inadequate control of RA symptoms [5 mg to 10 mg BID] or decreased in response to adverse events [AEs] or laboratory anomalies [10 mg to 5 mg BID]).

Patients receiving tofacitinib were also eligible for temporary discontinuation if deemed necessary by the investigator. In addition, patients participating in a vaccine sub-study (data not reported) underwent temporary withdrawal from tofacitinib treatment.

Patients were allowed to continue or add stable background arthritis therapy (including non-steroidal anti-inflammatory drugs, COX2 inhibitors, and opioids at ≤ 30 mg oral morphine/day potency), certain csDMARDs (MTX, leflunomide, sulfasalazine, anti-malarials, auranofin, and injectable gold preparations at approved doses), and corticosteroids (≤ 10 mg prednisone or equivalent/day), with adjustment permitted at the investigator’s discretion for reasons of inadequate efficacy, or tapering/discontinuation with disease improvement. Patients taking MTX must also have been taking folic acid (according to local standards). Prohibited concomitant medications included moderate/potent cytochrome P450 3A4 inhibitors or inducers.

This study was conducted in accordance with the International Ethical Guidelines for Biomedical Research Involving Human Subjects, the Declaration of Helsinki, and the Good Clinical Practice Guidelines, along with applicable local regulatory requirements and laws. The study protocol was approved by the Institutional Review Boards and/or Independent Ethics Committee at each study center. An independent Data Safety Monitoring Board external to the study sponsor reviewed unblinded safety data on a cumulative basis, and Safety Endpoint Adjudication Committees, blinded to treatment assignment of prior randomized double-blind index studies, supported with standardized safety endpoint assessment for selected events as described below. All patients provided written, informed consent.

### Patients

Patients who met the criteria for the index studies, which included a diagnosis of RA based on the American College of Rheumatology (ACR) 1987 Revised Criteria, and had completed the index studies were eligible for enrollment in ORAL Sequel. For those enrolling > 14 days after completion of their prior qualifying index study, the investigator must have deemed that their RA disease activity warranted treatment with tofacitinib and that no evidence of active or inadequately treated *Mycobacterium tuberculosis* infection be present. Key exclusion criteria for this LTE study included the following for all patients enrolling from the index studies: current/recent history of uncontrolled clinically significant renal, hepatic, hematologic, gastrointestinal, endocrine, pulmonary, cardiac, or neurologic disease; lifetime history of lymphoproliferative disorder, lymphoma, leukemia, or signs and symptoms suggestive of lymphatic disease; history of recurrent herpes zoster infection, current human immunodeficiency virus or hepatitis B/C infection, or any infection requiring hospitalization (including herpes zoster), parenteral antimicrobial therapy, or judged to be opportunistic by the investigator within 6 months prior to the first study drug dose (including those that occurred during the prior qualifying index study); current or history of malignancy (with the exception of adequately treated or excised non-metastatic basal/squamous cell skin cancer or cervical carcinoma in situ); or use of prohibited concomitant medications. For patients enrolling > 14 days after completion of their prior qualifying index study, additional exclusion criteria included hemoglobin levels < 9 g/dL or hematocrit < 30%; absolute white blood cell count < 3.0 × 10^9^/L, absolute neutrophil count (ANC) < 1.2 × 10^9^/L, or absolute lymphocyte count (ALC) < 0.5 × 10^9^/L (< 0.75 × 10^9^/L for patients in Croatia, Czech Republic, Denmark, Ireland, Korea, Germany, Spain, Sweden, and the UK); thrombocytopenia (platelet count < 100 × 10^9^/L); estimated creatinine clearance < 40 mL/min (Cockcroft-Gault calculation); and total bilirubin, aspartate aminotransferase (AST), or alanine aminotransferase (ALT) > 1.5 times the upper limit of normal (ULN).

### Objectives and endpoints

The primary objective was to determine the long-term safety and tolerability of tofacitinib 5 mg and 10 mg BID, via evaluation of AE reports, clinical laboratory data, physical examinations, vital signs, and electrocardiogram (ECG) values. The key secondary objective was to evaluate the long-term persistence of efficacy with tofacitinib 5 mg and 10 mg BID, via endpoints including ACR20/50/70 response rates; observed mean score over time in Health Assessment Questionnaire-Disability Index (HAQ-DI) and proportion of patients reporting improvements ≥ the minimum clinically important difference (≥ 0.22) in HAQ-DI; observed mean score over time in Disease Activity Score in 28 joints using erythrocyte sedimentation rate (DAS28-4[ESR]), and proportions of patients achieving DAS28-4(ESR)-defined remission (scores < 2.6) and DAS28-4(ESR)-defined low disease activity (LDA; scores ≤ 3.2); and the observed proportions of patients achieving remission defined by Clinical (CDAI) and Simplified (SDAI) Disease Activity Indices (scores ≤ 2.8 and ≤ 3.3, respectively). Exploratory ACR20/50/70 response maintenance analyses and CDAI/SDAI remission maintenance analyses were also conducted.

### Statistical methods

Statistical analyses of safety and efficacy data are descriptive in nature, and no formal comparisons between tofacitinib treatment groups were performed.

Baseline values for safety and efficacy endpoints were those of the qualifying index study (index baseline) for patients who enrolled in the LTE study within ≤ 14 days of index study completion, or the LTE baseline for patients who enrolled > 14 days after index study completion. The safety analysis set comprised all patients who received at least one dose of study medication.

Assignment to the tofacitinib 5 mg or 10 mg BID arm for analysis of efficacy and safety outcomes in this LTE study was based on the study average total daily dose (TDD) for each patient (i.e., sum of all doses received divided by number of days of treatment over the entire study duration for each patient): tofacitinib 5 mg BID if TDD < 15 mg and tofacitinib 10 mg BID if TDD ≥ 15 mg. Patients were assigned to “stay-on monotherapy” (received tofacitinib monotherapy) or “stay-on background csDMARDs” (received tofacitinib plus csDMARD combination therapy) subgroups for analysis of safety outcomes if they remained on their initial study-start therapy for the entire duration of the study (irrespective of tofacitinib or csDMARD dose adjustments), with the exception of a ≤ 28-day break in csDMARD use allowed for stay-on csDMARD patients. Patients who switched from csDMARD to monotherapy, or vice versa, were not included in these subgroup analyses and, therefore, they do not sum to total number of patients treated with tofacitinib. Equivalent subgroup analyses for efficacy outcomes, including data pooled from ORAL Sequel, have been previously published [17].

Exposure-adjusted event rates of the number of patients with events per 100 patient-years (EAERs per 100 patient-years) were calculated for AEs, and incidence rates of the number of patients with events per 100 patient-years (IRs per 100 patient-years) and 95% confidence intervals (CI; calculated via the Exact Poisson method) were calculated for AEs of special interest. EAERs were based on the number of unique patients with events per 100 patient-years over all patients’ exposures between their first dose of tofacitinib and their last dose (excluding any temporary treatment breaks in between). IRs were based on the number of unique patients with events during the time between the first and last tofacitinib dose plus 28 days, divided by the time accruing during the risk period (i.e., between the first and last tofacitinib dose plus 28 days, or the time accruing to the first event, whichever occurred earlier). The recurrence rate of herpes zoster was also calculated for patients with at least one event within the risk period.

In relation to IRs for AEs of special interest, cardiovascular events were adjudicated from February 2009, opportunistic infections from February 2013, hepatic events from December 2012, gastrointestinal events from December 2014, and interstitial lung disease events from April 2014. Events prior to these dates were not adjudicated and were identified by clinical review of AEs. For malignancies, the central histopathological review of AEs was initiated in July 2009, with events adjudicated from February 2014. Events prior to this were subsequently reviewed and adjudicated.

The efficacy analyses were conducted for all patients who received at least one dose of study medication and had at least one post-index/LTE baseline efficacy measurement available. All analyses were based on observed data with no imputation for missing data.

## Results

### Patients

Between February 5, 2007, and November 30, 2016, 4481 patients were enrolled in the main study of ORAL Sequel (Additional file 2: Figure S1a). A total of 2340 patients discontinued (see Kaplan-Meier time to discontinuation curve in Additional file 2: Figure S1b). For all tofacitinib, the median time to discontinuation was 1785 days (approximately 4.9 years), as estimated by the Kaplan-Meier method. After 5 years, 49% of patients were still in the study.

Patient baseline demographic and disease characteristics (Additional file 3: Table S2) were generally similar between treatment arms. In total, 90.2% (*n* = 4041/4481) of patients had baseline data from their index study (i.e., patients enrolled ≤ 14 days after index study completion) and 9.8% (*n* = 440/4481) had baseline data re-assessed and reported at the time of enrollment into the LTE study (i.e., patients enrolled > 14 days after index study completion).

The majority of patients (76.6% [3432/4481]) remained on their initial tofacitinib dose (5 mg or 10 mg BID) throughout the study.

### Safety

#### All-cause adverse events

AE data are presented for all patients up to month 114 of the LTE period for tofacitinib 5 mg and 10 mg BID. Total tofacitinib exposure was 16,291 patient-years (4683 patient-years in the 5 mg BID population and 11,608 patient-years in the 10 mg BID population). A summary of all-cause treatment-emergent AEs (hereafter referred to as AEs), including the most common all-cause AEs by system organ class (SOC) and preferred term, is presented in Table 1. The majority of all-cause AEs were mild (59%) or moderate (36%) in severity for all tofacitinib; corresponding data for tofacitinib 5 mg BID were 57% and 36%, respectively, and for tofacitinib 10 mg BID were 59% and 36%, respectively.

**Table 1:** Patients with all-cause treatment-emergent AEs

	Tofacitinib 5 mg BID (*n* = 1123)	Tofacitinib 10 mg BID (*n* = 3358)	All tofacitinib (*n* = 4481)
AEs, *n* (%) [IRs; patients with events per 100 patient-years; 95% CI]			
AEs	1015 (90.4) [98.69; 92.71, 104.96]	3021 (90.0) [118.54; 114.35, 122.84]	4036 (90.1) [112.83; 109.38, 116.37]
SAEs	346 (30.8) [8.16; 7.31, 9.07]	997 (29.7) [9.37; 8.80, 9.98]	1343 (30.0) [9.03; 8.55, 9.53]
Discontinued due to AEs	315 (28.0) [6.67; 5.95, 7.45]	805 (24.0) [6.83; 6.36, 7.32]	1120 (25.0) [6.78; 6.39, 7.20]
Dose reduction or temporary discontinuation due to AEs	518 (46.1) [16.85; 15.43, 18.36]	1329 (39.6) [15.70; 14.87, 16.57]	1847 (41.2) [16.01; 15.29, 16.76]
Dose reduction only	75 (14.5)	86 (6.5)	161 (8.7)
Temporary discontinuation only	372 (71.8)	1147 (86.3)	1519 (82.2)
Dose reduction and temporary discontinuation	71 (13.7)	96 (7.2)	167 (9.0)
Permanent discontinuation^a^	275 (53.1)	615 (46.3)	890 (48.2)
AEs, *n* (%) [EAERs; patients with events per 100 patient-years] Most frequently reported AEs by SOC (≥ 20% in any treatment group) and within-SOC preferred term (≥ 5% in any treatment group)			
Infections and infestations	738 (65.7) [15.90]	2299 (68.5) [20.04]	3037 (67.8) [18.84]
Upper respiratory tract infection	228 (20.3) [4.91]	614 (18.3) [5.35]	842 (18.8) [5.22]
Nasopharyngitis	138 (12.3) [2.97]	518 (15.4) [4.51]	656 (14.6) [4.07]
Urinary tract infection	166 (14.8) [3.57]	453 (13.5) [3.94]	619 (13.8) [3.84]
Bronchitis	143 (12.7) [3.08]	434 (12.9) [3.78]	577 (12.9) [3.58]
Herpes zoster	119 (10.6) [2.56]	386 (11.5) [3.36]	505 (11.3) [3.13]
Sinusitis	70 (6.2) [1.50]	242 (7.2) [2.10]	312 (7.0) [1.93]
Influenza	81 (7.2) [1.74]	199 (5.9) [1.73]	280 (6.2) [1.73]
Pharyngitis	58 (5.2) [1.24]	148 (4.4) [1.29]	206 (4.6) [1.27]
Musculoskeletal and connective tissue disorders	447 (39.8) [9.63]	1373 (40.9) [11.96]	1820 (40.6) [11.29]
Rheumatoid arthritis	111 (9.9) [2.39]	309 (9.2) [2.69]	420 (9.4) [2.60]
Back pain	109 (9.7) [2.34]	301 (9.0) [2.62]	410 (9.1) [2.54]
Arthralgia	88 (7.8) [1.89]	271 (8.1) [2.36]	359 (8.0) [2.22]
Osteoarthritis	66 (5.9) [1.42]	196 (5.8) [1.70]	262 (5.8) [1.62]
Gastrointestinal disorders	406 (36.2) [8.74]	1045 (31.1) [9.10]	1451 (32.4) [9.00]
Diarrhea	74 (6.6) [1.59]	218 (6.5) [1.90]	292 (6.5) [1.81]
Nausea	57 (5.1) [1.22]	175 (5.2) [1.52]	232 (5.2) [1.43]
Investigations	404 (36.0) [8.70]	1007 (30.0) [8.77]	1411 (31.5) [8.75]
Blood creatine phosphokinase increased	91 (8.1) [1.96]	249 (7.4) [2.17]	340 (7.6) [2.11]
ALT increased	63 (5.6) [1.35]	122 (3.6) [1.06]	185 (4.1) [1.14]
Blood creatinine increased	60 (5.3) [1.29]	116 (3.5) [1.01]	176 (3.9) [1.09]
Injury, poisoning, and procedural complications	267 (23.8) [5.75]	783 (23.3) [6.82]	1050 (23.4) [6.51]
Fall	70 (6.2) [1.50]	217 (6.5) [1.89]	287 (6.4) [1.78]
Nervous system disorders	232 (20.7) [4.99]	687 (20.5) [5.98]	919 (20.5) [5.70]
Headache	67 (6.0) [1.44]	203 (6.0) [1.76]	270 (6.6) [1.67]
Respiratory, thoracic, and mediastinal disorders	223 (19.9) [4.80]	671 (20.0) [5.84]	894 (20.0) [5.54]
Cough	65 (5.8) [1.40]	203 (6.0) [1.76]	268 (6.0) [1.66]

Safety analysis set: all patients who received at least one dose of study medication. Database lock: March 2, 2017

*AE* adverse event, *ALT* alanine aminotransferase, *BID* twice daily, *CI* confidence interval, *EAER* exposure-adjusted event rate, *IR* incidence rate, *SAE* serious adverse event, *SOC* system organ class

^a^Patients who had dose reduction or temporary discontinuation due to AEs and eventually discontinued from the study

EAERs represent rates of events/100 patient-years of exposure. Total tofacitinib exposure was 16,291 patient-years (4683 patient-years in the 5 mg BID population and 11,608 patient-years in the 10 mg BID population). Exposure for EAER calculations was 16,113 patient-years for all tofacitinib (4641 patient-years in the 5 mg BID population and 11,472 patient-years in the 10 mg BID population). Data for herpes zoster reflect only AEs reported using preferred term “Herpes zoster”

#### All-cause adverse events leading to discontinuation

The proportion of patients with all-cause AEs decreased from baseline over time, while the proportion with all-cause AEs leading to discontinuation remained consistent over time (Additional file 4: Figure S2). For all tofacitinib, the most common all-cause AEs by SOC leading to discontinuation included infections and infestations (9.4% [*n* = 423/4481]), investigations (4.6% [*n* = 206/4481]), and benign, malignant, and unspecified neoplasms (3.7% [*n* = 165/4481]), and by preferred term included pneumonia (1.8% [*n* = 80/4481]), blood creatinine increased (1.5% [*n* = 69/4481]), and herpes zoster (0.7% [*n* = 32/4481]). The IR (95% CI) for all-cause AEs leading to discontinuation was 6.78 (6.39, 7.20) for all tofacitinib. The corresponding IR data for patients receiving tofacitinib as combination therapy (*n* = 656/2464) was 7.73 (7.15, 8.35), and for patients receiving tofacitinib as monotherapy (*n* = 279/1298) was 5.88 (5.21, 6.61).

#### Serious adverse events

For all tofacitinib, the most common (≥ 5% in any treatment group) all-cause serious AEs (SAEs) by SOC included infections and infestations (9.0% [*n* = 405/4481]) and musculoskeletal and connective tissue disorders (5.5% [*n* = 246/4481]), and by preferred term included pneumonia (2.1% [*n* = 96/4481]), osteoarthritis (1.9% [*n* = 86/4481]), and RA (0.8% [*n* = 34/4481]), noting that 1.1% (*n* = 51/4481) also reported “condition aggravated” as an SAE. The IR (95% CI) for SAEs was 9.03 (8.55, 9.53) for all tofacitinib. The corresponding SAE IR (95% CI) for patients receiving tofacitinib as combination therapy (*n* = 726/2464) was 9.48 (8.80, 10.20), and for patients receiving tofacitinib as monotherapy (*n* = 349/1298) was 8.11 (7.28, 9.01).

#### Deaths

A total of 88 deaths occurred in the study; 44 within the risk period (excluding one fetal death [pregnancy in partner of enrolled patient with in utero fetal death reported after diagnosis of Down syndrome]) and 43 outside of the risk period. A total number of 84 all-cause SAEs (5 mg BID *n* = 39, 10 mg BID *n* = 45) resulted in death. The IR (95% CI) for all-cause mortality was 0.26 (0.19, 0.36) for all tofacitinib. The corresponding all-cause mortality IR (95% CI) for patients receiving tofacitinib as combination therapy (*n* = 23/2464) was 0.27 (0.17, 0.40), and for patients receiving tofacitinib as monotherapy (*n* = 18/1298) was 0.37 (0.22, 0.59). Further details (incidence of mortality and mortality listings) are provided in Additional file 5: Table S3.

#### All-cause adverse events of special interest

**Fig. 1:**
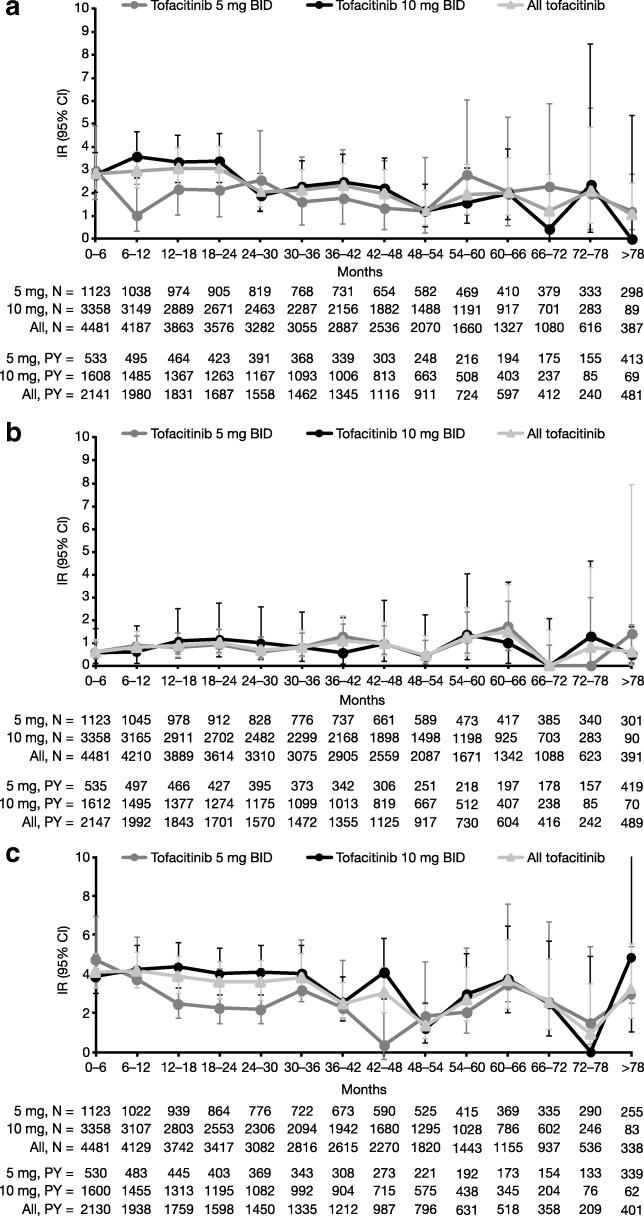
Incidence rates for notable safety events of special interest over time. Including serious infections (**a**), malignancies excluding NMSC (**b**), and herpes zoster (**c**). IR is the number of patients with events per 100 patient-years. Total tofacitinib exposure was 16,291 patient-years (4683 patient-years in the 5 mg BID population and 11,608 patient-years in the 10 mg BID population). Database lock: March 2, 2017. *BID* twice daily, *CI* confidence interval, *IR* incidence rate, *NMSC* non-melanoma skin cancer, *PY* patient-years of exposure

IRs for all-cause AEs of special interest are summarized in Table 2, including data for patients receiving tofacitinib as combination therapy and as monotherapy. The majority of malignancy-, cardiovascular-, mortality-, and infection-related events had IRs < 0.5; exceptions were herpes zoster, all serious infections, malignancies excluding non-melanoma skin cancer (NMSC), and NMSC. Figure 1a–c presents serious infections, malignancies excluding NMSC, and herpes zoster over time, showing they remained generally stable. IRs for malignancies excluding NMSC, lymphoma, melanomas, breast cancer (female patients only), lung cancer, tuberculosis, composite major adverse cardiovascular event (MACE), gastrointestinal perforation, interstitial lung disease, deep vein thrombosis (DVT), pulmonary embolism (PE), and mortality were comparable between patients receiving tofacitinib 5 mg and 10 mg BID.

**Table 2:** Incidence rates for all-cause AEs of special interest

	All patients (IR (95% CI) [*n*/*N*])			Patients receiving tofacitinib as combination therapy (stay-on background csDMARDs) (IR (95% CI) [*n*/*N*])			Patients receiving tofacitinib as monotherapy (stay-on monotherapy) (IR (95% CI) [*n*/*N*])		
	Tofacitinib 5 mg BID	Tofacitinib 10 mg BID	All tofacitinib	Tofacitinib 5 mg BID	Tofacitinib 10 mg BID	All tofacitinib	Tofacitinib 5 mg BID	Tofacitinib 10 mg BID	All tofacitinib
Malignancy related									
NMSC	0.6 (0.4, 0.9) [28/1123]	0.8 (0.6, 0.9) [88/3358]	0.7 (0.6, 0.9) [116/4481]	0.4 (0.2, 0.7) [9/630]	0.9 (0.6, 1.1) [51/1834]	0.7 (0.5, 0.9) [60/2464]	0.8 (0.4, 1.4) [10/305]	0.4 (0.2, 0.6) [13/993]	0.5 (0.3, 0.7) [23/1298]
Malignancies excluding NMSC	0.8 (0.6, 1.1) [38/1123]	0.8 (0.7, 1.0) [100/3358]	0.8 (0.7, 1.0) [138/4481]	1.0 (0.7, 1.5) [25/630]	0.8 (0.6, 1.0) [48/1834]	0.9 (0.7, 1.1) [73/2464]	0.8 (0.4, 1.4) [10/305]	0.9 (0.6, 1.3) [31/993]	0.9 (0.6, 1.2) [41/1298]
Lymphoma	0.0 (0.0, 0.1) [1/1123]	0.1 (0.0, 0.1) [8/3358]	0.1 (0.0, 0.1) [9/4481]	0.0 (0.0, 0.2) [1/630]	0.1 (0.0, 0.2) [5/1834]	0.1 (0.0, 0.2) [6/2464]	0 (0.0, 0.3) [0/305]	0.0 (0.0, 0.2) [1/993]	0.0 (0.0, 0.1) [1/1298]
Melanomas	0.1 (0.0, 0.2) [3/1123]	0.1 (0.0, 0.2) [10/3358]	0.1 (0.0, 0.1) [13/4481]	0.1 (0.0, 0.3) [2/630]	0.1 (0.0, 0.2) [4/1834]	0.1 (0.0, 0.2) [6/2464]	0.1 (0.0, 0.4) [1/305]	0.1 (0.1, 0.3) [5/993]	0.1 (0.1, 0.3) [6/1298]
Breast cancer^a^	0.2 (0.1, 0.3) [6/927]	0.2 (0.1, 0.3) [17/2744]	0.2 (0.1, 0.3) [23/3671]	0.3 (0.1, 0.6) [5/512]	0.2 (0.1, 0.4) [10/1498]	0.2 (0.1, 0.4) [15/2010]	0 (0.0, 0.3) [0/261]	0.1 (0.0, 0.3) [3/798]	0.1 (0.0, 0.2) [3/1059]
Lung cancer	0.1 (0.1, 0.3) [6/1123]	0.2 (0.1, 0.3) [20/3358]	0.2 (0.1, 0.2) [26/4481]	0.1 (0.0, 0.4) [3/630]	0.2 (0.1, 0.3) [12/1834]	0.2 (0.1, 0.3) [15/2464]	0.2 (0.0, 0.6) [2/305]	0.2 (0.1, 0.4) [6/993]	0.2 (0.1, 0.3) [8/1298]
Infection related									
All serious infections	1.9 (1.6, 2.4) [91/1123]	2.6 (2.3, 2.9) [304/3358]	2.4 (2.2, 2.6) [395/4481]	1.9 (1.4, 2.5) [46/630]	3.0 (2.6, 3.5) [184/1834]	2.7 (2.4, 3.1) [230/2464]	2.1 (1.4, 3.0) [27/305]	2.3 (1.9, 2.9) [81/993]	2.3 (1.9, 2.7) [108/1298]
Tuberculosis	0.1 (0.0, 0.2) [5/1123]	0.2 (0.1, 0.3) [20/3358]	0.2 (0.1, 0.2) [25/4481]	0.2 (0.0, 0.4) [4/630]	0.2 (0.1, 0.3) [12/1834]	0.2 (0.1, 0.3) [16/2464]	0.1 (0.0, 0.4) [1/305]	0.2 (0.1, 0.4) [6/993]	0.2 (0.1, 0.3) [7/1298]
Herpes zoster	2.3 (2.3, 3.3) [120/1123]	3.7 (3.4, 4.1) [406/3358]	3.4 (3.2, 3.7) [526/4481]	2.2 (1.7, 2.9) [52/630]	4.1 (3.6, 4.7) [233/1834]	3.6 (3.2, 4.0) [285/2464]	2.4 (1.6, 3.5) [29/305]	2.3 (1.8, 2.9) [77/993]	2.4 (1.9, 2.8) [106/1298]
Opportunistic infections excluding tuberculosis	0.1 (0.0, 0.2) [4/1123]	0.5 (0.4, 0.7) [60/3358]	0.4 (0.3, 0.5) [64/4481]	0.0 (0.0, 0.2) [1/630]	0.6 (0.4, 0.8) [36/1834]	0.4 (0.3, 0.6) [37/2464]	0.2 (0.0, 0.6) [2/305]	0.4 (0.2, 0.7) [14/993]	0.3 (0.2, 0.5) [16/1298]
Mortality related									
Mortality attributed to a cardiovascular event	0.2 (0.1, 0.3) [7/1046]	0.1 (0.1, 0.2) [12/3358]	0.1 (0.1, 0.2) [19/4404]	0.1 (0.0, 0.4) [3/572]	0.1 (0.1, 0.3) [8/1834]	0.1 (0.1, 0.2) [11/2406]	0.3 (0.1, 0.8) [4/289]	0.1 (0.0, 0.2) [2/993]	0.1 (0.1, 0.3) [6/1282]
Mortality due to infections	0.1 (0.0, 0.2) [4/1123]	0.1 (0.0, 0.1) [9/3358]	0.1 (0.0, 0.1) [13/4481]	0.1 (0.0, 0.3) [2/630]	0.1 (0.0, 0.2) [4/1834]	0.1 (0.0, 0.2) [6/2464]	0.1 (0.0, 0.4) [1/305]	0.1 (0.0, 0.3) [4/993]	0.1 (0.0, 0.2) [5/1298]
Mortality due to malignancy	0.0 (0.0, 0.1) [1/1123]	0.0 (0.0, 0.1) [3/3358]	0.0 (0.0, 0.1) [4/4481]	0 (0.0, 0.2) [0/630]	0.0 (0.0, 0.1) [2/1834]	0.0 (0.0, 0.1) [2/2464]	0.1 (0.0, 0.4) [1/305]	0.0 (0.0, 0.2) [1/993]	0.0 (0.0, 0.2) [2/1298]
Other									
Composite MACE	0.5 (0.3, 0.7) [20/1046]	0.4 (0.3, 0.5) [42/3358]	0.4 (0.3, 0.5) [62/4404]	0.5 (0.3, 0.9) [11/572]	0.5 (0.3, 0.7) [29/1834]	0.5 (0.4, 0.7) [40/2406]	0.5 (0.2, 1.1) [6/289]	0.2 (0.1, 0.4) [6/993]	0.3 (0.1, 0.4) [12/1282]
Gastrointestinal perforation	0.1 (0.0, 0.2) [3/1123]	0.2 (0.1, 0.3) [20/3358]	0.1 (0.1, 0.2) [23/4481]	0.0 (0.0, 0.2) [1/630]	0.2 (0.1, 0.4) [13/1834]	0.2 (0.1, 0.3) [14/2464]	0.1 (0.0, 0.4) [1/305]	0.1 (0.1, 0.3) [5/993]	0.1 (0.1, 0.3) [6/1298]
Interstitial lung disease	0.2 (0.1, 0.4) [10/1123]	0.2 (0.1, 0.3) [22/3358]	0.2 (0.1, 0.3) [32/4481]	0.2 (0.0, 0.4) [4/630]	0.3 (0.1, 0.4) [15/1834]	0.2 (0.1, 0.4) [19/2464]	0.3 (0.1, 0.8) [4/305]	0.1 (0.0, 0.3) [3/993]	0.2 (0.1, 0.3) [7/1298]
Deep vein thrombosis	0.1 (0.1, 0.3) [6/1123]	0.1 (0.1, 0.2) [17/3358]	0.1 (0.1, 0.2) [23/4481]	0.1 (0.0, 0.4) [3/630]	0.1 (0.1, 0.3) [8/1834]	0.1 (0.1, 0.2) [11/2464]	0.1 (0.0, 0.4) [1/305]	0.2 (0.1, 0.4) [6/993]	0.2 (0.1, 0.3) [7/1298]
Pulmonary embolism	0.1 (0.0, 0.3) [5/1123]	0.1 (0.1, 0.2) [16/3358]	0.1 (0.1, 0.2) [21/4481]	0.1 (0.0, 0.3) [2/630]	0.1 (0.1, 0.3) [8/1834]	0.1 (0.1, 0.2) [10/2464]	0.1 (0.0, 0.4) [1/305]	0.2 (0.1, 0.4) [6/993]	0.2 (0.1, 0.3) [7/1298]

IR is the number of patients with events per 100 patient-years. Tofacitinib was dosed at 1, 3, 5, 10, 15, and 30 mg BID, or 20 mg QD, as monotherapy or in combination with background csDMARDs (mostly MTX). Total tofacitinib exposure was 16,291 patient-years (4683 patient-years in the 5 mg BID population and 11,608 patient-years in the 10 mg BID population). Tofacitinib exposure for female patients was 13,476 patient-years (3888 patient-years in the 5 mg BID population and 9587 patient-years in the 10 mg BID population). Tofacitinib exposure for patients in the cardiac event analysis population was 15,823 patient-years (4257 patient-years in the 5 mg BID population and 11,566 patient-years in the 10 mg BID population). As per the protocol, cardiovascular events were adjudicated from February 2009, opportunistic infections from February 2013, hepatic events from December 2012, gastrointestinal events from December 2014, and interstitial lung disease events from April 2014. Events prior to these dates were not adjudicated and were identified by clinical review of AEs. For malignancies, central histopathological review of AEs was initiated in July 2009, with events adjudicated from February 2014. Events prior to this were subsequently reviewed and adjudicated. Data for herpes zoster reflect AEs reported using any preferred term including “Herpes zoster”. Database lock: March 2, 2017

*AE* adverse event, *BID* twice daily, *CI* confidence interval, *csDMARD* conventional synthetic disease-modifying antirheumatic drug, *IR* incidence rate, *MACE* major adverse cardiovascular event, *MTX* methotrexate, *NMSC* non-melanoma skin cancer, *QD* once daily

^a^Female patients only

Across all patients, IRs were higher (per non-overlapping CI) with tofacitinib 10 mg BID versus 5 mg BID for herpes zoster (3.7 versus 2.3, respectively) and opportunistic infections excluding tuberculosis (0.5 versus 0.1); numerical differences (per marginally overlapping CI) in IRs were also observed for all serious infections (Table 2). The majority of cases of herpes zoster were non-serious (96% [503/526]); 30 patients had recurrent herpes zoster, including 1 patient with recurrent ophthalmic herpes zoster. The recurrence rate for herpes zoster (per 100 patient-years) was 2.6.

In addition, IRs were higher for patients receiving tofacitinib as combination therapy versus monotherapy for herpes zoster (3.6 versus 2.4, respectively). Within the subgroup of patients receiving tofacitinib as combination therapy, IRs were higher with tofacitinib 10 mg BID versus 5 mg BID for all serious infections (3.0 versus 1.9, respectively), herpes zoster (4.1 versus 2.2), and opportunistic infections excluding tuberculosis (0.6 versus 0.0) (Table 2).

#### Laboratory variables of interest

**Fig. 2:**
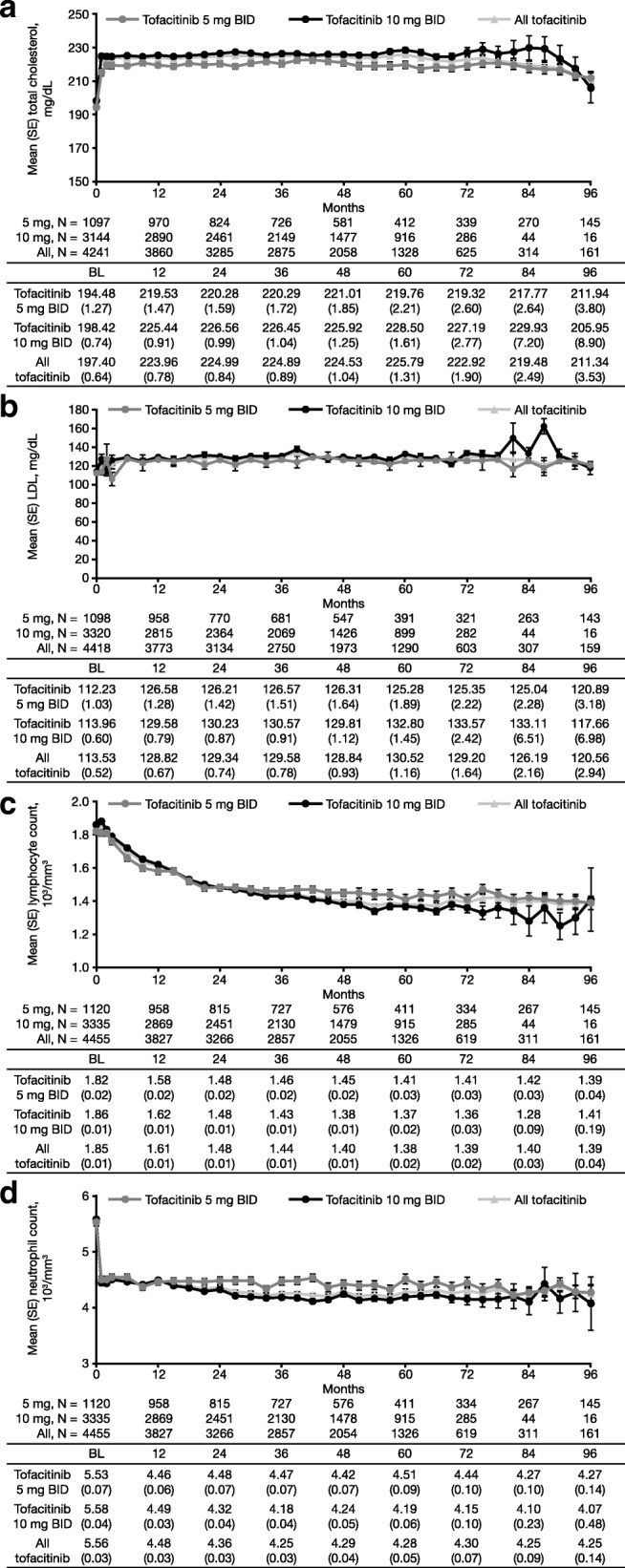
Mean (SE) laboratory variables of interest over time. Including total cholesterol (**a**), LDL (**b**), lymphocyte counts (**c**), and neutrophil counts (**d**). Baseline qualifying index study data were used for approximately 90% of patients. Data for 12-month intervals are reported in the tables. Database lock: March 2, 2017. *BID* twice daily, *BL* baseline, *LDL* low-density cholesterol, *SE* standard error

Laboratory variables of interest, including total cholesterol and low-density lipoprotein (LDL) (Fig. 2a, b), ALT (Additional file 6: Figure S3a), AST (Additional file 6: Figure S3b), and serum creatinine (Additional file 6: Figure S3d) remained generally stable over time, with variability attributable to smaller patient numbers at later time points. Changes over time were observed in lymphocytes (ALC levels gradually declined until month 48, whereafter the level stabilized; Fig. 2c) and neutrophils (Fig. 2d); furthermore, slight increases in hemoglobin (Additional file 6: Figure S3c) were observed until month 24, which then remained stable. Laboratory data are reported up to month 96 of the LTE study period only, due to low patient numbers after this time point.

For all tofacitinib, values for confirmed neutropenia at any time were mild, 1.5 × 10^3^/mm^3^ ≤ ANC < 2 × 10^3^/mm^3^, 6.0% (271/4481); moderate, 1.0 × 10^3^/mm^3^ ≤ ANC < 1.5 × 10^3^/mm^3^, 1.3% (58/4481); severe, 0.5 × 10^3^/mm^3^ ≤ ANC < 1.0 × 10^3^/mm^3^, 0.2% (7/4481); and potentially life-threatening, ANC < 0.5 × 10^3^/mm^3^, 0%). No patients with confirmed neutropenia developed serious infections within 30 days of their lowest neutrophil count. For confirmed lymphopenia at any time, values were mild, 1.5 × 10^3^/mm^3^ ≤ ALC < 2 × 10^3^/mm^3^, 17.5% (782/4481); moderate, 1.0 × 10^3^/mm^3^ ≤ ALC < 1.5 × 10^3^/mm^3^, 40.6% (1820/4481); severe, 0.5 × 10^3^/mm^3^ ≤ ALC < 1.0 × 10^3^/mm^3^, 30.1% (1351/4481); and potentially life-threatening, ALC < 0.5 × 10^3^/mm^3^, 1.3% (58/4481). Of the patients with mild, moderate, and severe lymphopenia, 0.9% (7/782), 0.3% (5/1820), and 0.3% (4/1351), respectively, developed serious infections within 30 days of their lowest ALC. Of the 58 patients with potentially life-threatening lymphopenia at any time (with ALC < 0.5 × 10^3^ cells/mm^3^), five cases were potentially associated with serious infections; two (3.4%) occurred within 30 days of the patients’ lowest ALC. In addition, 52 of the 58 patients eventually returned to ALC ≥ 0.5 × 10^3^ cells/mm^3^, and 29 of the 58 patients with ALC < 0.5 × 10^3^ cells/mm^3^ eventually returned to within 20% of their respective baseline value, following treatment discontinuation. For all tofacitinib, the IRs (95% CI) for neutropenia (*n* = 86/4481) and lymphopenia (*n* = 181/4481) were 0.52 (0.42, 0.65) and 1.11 (0.95, 1.28), respectively.

The proportions of patients with confirmed ALT and AST > 1×, ≥ 2×, and ≥ 3× ULN are shown in Additional file 7: Table S4. The IR (95% CI) for ALT ≥ 3× ULN was 1.71 (1.51, 1.92) for all tofacitinib; the corresponding IR for patients receiving tofacitinib as combination therapy (*n* = 133/2464) was 1.59 (1.33, 1.88), and for patients receiving tofacitinib as monotherapy (*n* = 70/1298) was 1.50 (1.17, 1.90). For AST ≥ 3× ULN, IRs (95% CI) were 0.98 (0.84, 1.15), 1.04 (0.83, 1.28), and 0.93 (0.68, 1.25) for all tofacitinib, patients receiving tofacitinib as combination therapy (*n* = 88/2464), and patients receiving tofacitinib as monotherapy (*n* = 44/1298), respectively.

The summary of actions taken with tofacitinib and MTX during elevated AST or ALT levels ≥ 3× ULN is shown in Table 3.

**Table 3:** Summary of actions taken with tofacitinib and MTX during ALT or AST elevations ≥ 3× ULN

Patients with ≥ 3× ULN ALT or AST (*N* = 109), *n* (%)						
Actions taken with MTX	Actions taken with tofacitinib					
	Continued tofacitinib (*n* = 40)	Permanently discontinued tofacitinib (*n* = 39)	Discontinued tofacitinib due to AEs^a^ (*n* = 2)	Temporarily discontinued tofacitinib and resumed same dose (*n* = 10)	Temporarily discontinued tofacitinib and resumed reduced dose^b^ (*n* = 7)	Reduced tofacitinib dose^b^ (*n* = 11)
Permanently discontinued MTX	4 (3.7)	14 (12.8)	1 (0.9)	1 (0.9)	0	2 (1.8)
Temporarily discontinued MTX	1 (0.9)	0	0	0	0	0
Reduced MTX dose	2 (1.8)	0	0	0	1 (0.9)	0
Continued MTX	20 (18.3)	11 (10.1)	0	4 (3.7)	4 (3.7)	2 (1.8)
Patients were not receiving MTX	13 (11.9)	14 (12.8)	1 (0.9)	5 (4.6)	2 (1.8)	7 (6.4)

*AE* adverse event, *ALT* alanine aminotransferase, *AST* aspartate aminotransferase, *BID* twice daily, *MTX* methotrexate, *ULN* upper limit of normal

^a^AEs occurring prior to AST elevations

^b^Reduction in tofacitinib dose from 10 mg BID to 5 mg BID

For all tofacitinib, laboratory values that met with protocol criteria for monitoring included any single ALT and/or AST elevation > 3× ULN regardless of total bilirubin (7.1% [317/4481]); any single hemoglobin value < 8.0 g/dL, or one that drops ≥ 2 gm/dL below baseline (14.3% [640/4481]); and transient increases in serum creatinine > 50% over the average screening and baseline values leading to discontinuation (4.5% [201/4481] patients). Of note, no patients had confirmed ANC < 500 mm^3^. No clinically notable changes were observed in systolic or diastolic blood pressure or ECG values, from baseline to month 96 (data not shown).

### Efficacy

Efficacy data are presented up to month 96 of the LTE study period for tofacitinib 5 mg BID, and up to month 72 of the LTE study period for tofacitinib 10 mg BID (data were censored due to low patient numbers after these time points; although these patients contributed to all tofacitinib exposure).

#### ACR

**Fig. 3:**
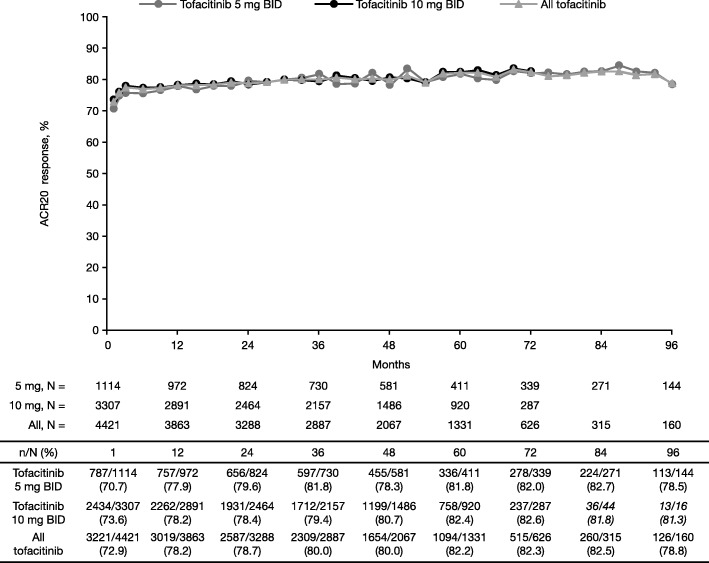
ACR20 response over time (observed). ACR calculated with respect to qualifying index study data available for approximately 90% of patients. Italicized data not reported in figure due to low patient numbers. Data for 12-month intervals are reported in the table. Database lock: March 2, 2017. *ACR* American College of Rheumatology, *BID* twice daily

ACR20 (Fig. 3), ACR50 (Additional file 8: Figure S4a), and ACR70 (Additional file 8: Figure S4b) response rates were maintained over time between months 1 and 96 and were generally similar with tofacitinib 5 mg (months 1 to 96) and 10 mg BID (months 1 to 72).

#### HAQ-DI

**Fig. 4:**
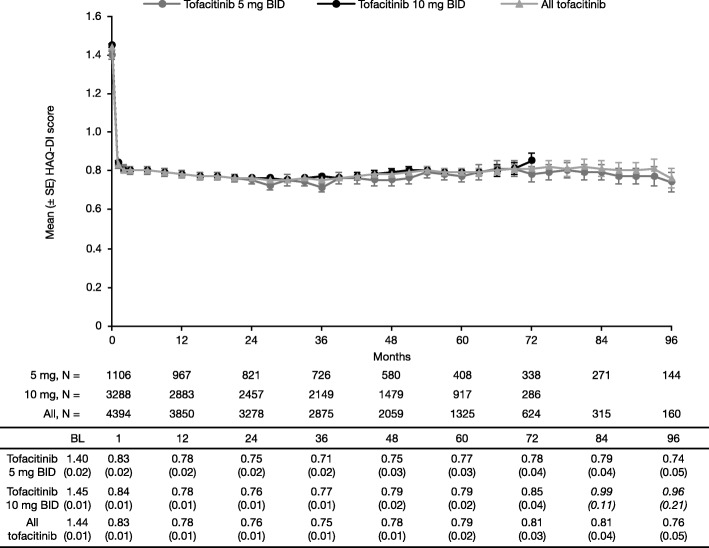
Mean (SE) HAQ-DI scores over time (observed). Baseline qualifying index study data were used for approximately 90% of patients. Italicized data not reported in figure due to low patient numbers. Data for 12-month intervals are reported in the table. Database lock: March 2, 2017. *BID* twice daily, *BL* baseline, *HAQ-DI* Health Assessment Questionnaire-Disability Index, *SE* standard error

Acknowledging appreciable attrition in the sample size over time, improvements in mean HAQ-DI scores at month 1 remained stable over time with tofacitinib 5 mg and 10 mg BID (Fig. 4). HAQ-DI ≥ 0.22 improvement from baseline was observed in 64.8% (*n* = 103/159) of patients with all tofacitinib at month 96; in 63.6% (*n* = 91/143) of patients with tofacitinib 5 mg BID at month 96; and in 70.3% (*n* = 201/286) of patients with tofacitinib 10 mg BID at month 72.

#### DAS28-4(ESR), CDAI, and SDAI

**Fig. 5:**
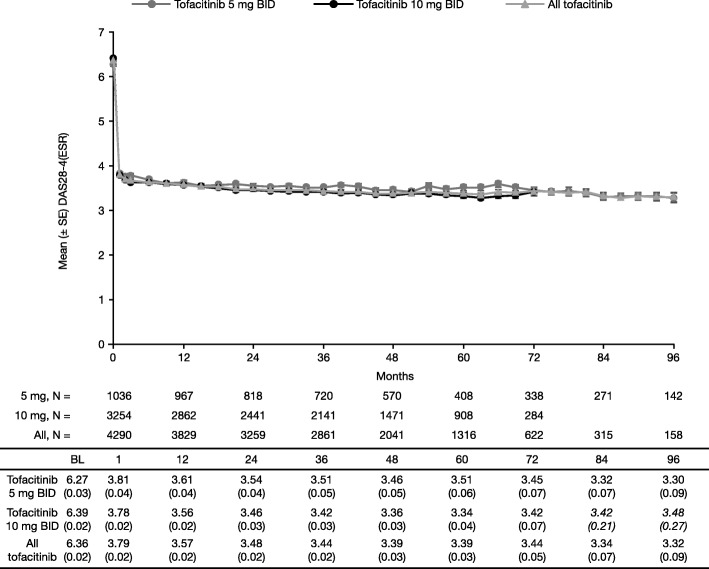
Mean (SE) DAS28-4(ESR) over time (observed). Baseline qualifying index study data were used for approximately 90% of patients. Italicized data not reported in figure due to low patient numbers. Data for 12-month intervals are reported in the table. Database lock: March 2, 2017. *BID* twice daily, *BL* baseline, *DAS28-4(ESR)* Disease Activity Score in 28 joints using erythrocyte sedimentation rate, *SE* standard error

Mean DAS28-4(ESR) (Fig. 5) decreased at month 1 and then remained consistent over time with tofacitinib 5 mg and 10 mg BID. DAS28-4(ESR)-defined remission was observed in 24.7% (*n* = 39/158) of patients with all tofacitinib at month 96, in 25.4% (*n* = 36/142) of patients with tofacitinib 5 mg BID at month 96, and in 25.0% (*n* = 71/284) of patients with tofacitinib 10 mg BID at month 72. Corresponding data for DAS28-4(ESR)-defined LDA were 46.8% (*n* = 74/158), 47.2% (*n* = 67/142), and 48.2% (*n* = 137/284), respectively.

CDAI- and SDAI-defined remission was observed in approximately a third of patients at month 96 (Additional file 9: Figure S5a and S5b). Rates of CDAI and SDAI remission were maintained over time, with the majority (> 85%) of patients maintaining or improving their CDAI or SDAI category (CDAI ≤ 2.8; 2.8 < CDAI ≤ 10; CDAI > 10) from the end of their index study to month 96 of the LTE.

## Discussion

The ORAL Sequel LTE study of tofacitinib is part of the largest clinical development program undertaken for any RA treatment to date. Up to 9.5 years of treatment with tofacitinib in more than 4000 patients worldwide with RA is represented in this study, with a combined tofacitinib exposure of > 16,000 patient-years.

Safety data up to 114 months for all tofacitinib, and efficacy data up to 96 months for tofacitinib 5 mg BID and 72 months for 10 mg BID, are reported, with low patient numbers limiting interpretation beyond these time points. Patient baseline demographic and disease characteristics were generally similar between the tofacitinib 5 mg and 10 mg BID treatment arms, although a greater proportion of patients enrolled from prior phase 3 studies compared with phase 2 studies and then initiated the LTE at 10 mg BID, per protocol (the exception to this was that all phase 3 patients from China initiated the LTE at 5 mg BID, also per protocol). Most (> 90%) patients were enrolled in the LTE study within ≤ 14 days of index study completion, and so their baseline index data were used, per protocol.

In all, the safety profile remained consistent with that observed in prior phase 2 [2–6] or phase 3 [7, 9–13] tofacitinib studies, or prior combined analyses including LTE data [16, 18]. The IRs for AEs leading to discontinuation were comparable for all tofacitinib, tofacitinib 5 mg BID, and tofacitinib 10 mg BID (6.8, 6.7, and 6.8, respectively), as were the IRs for SAEs (9.0, 8.2, and 9.4, respectively). Overall in ORAL Sequel, 52% of patients discontinued by month 114 (24% due to AEs and 4% due to insufficient clinical response) and the incidence of AEs leading to discontinuation remained stable over time. These data are consistent with a recent integrated analysis of safety data over 8.5 years for tofacitinib (5 mg and 10 mg BID combined, with or without background csDMARDs) across 17 phase 1/2/3 studies and two LTE studies, including ORAL Sequel, in which the IR for discontinuation due to AEs was 7.5 and the IR for SAEs was 9.0, for all tofacitinib [18]. Furthermore, in pooled LTE safety analyses from ORAL Sequel and Japanese Study A3921041 (tofacitinib 5 mg and 10 mg BID combined, with or without background DMARDs), 51% of patients discontinued by month 114 (24% due to AEs and 4% due to insufficient clinical response) and the IR for SAEs was 9.1 for all tofacitinib [16].

Most malignancy-, cardiovascular-, mortality-, and infection-related events had IRs < 0.5; exceptions were herpes zoster, all serious infections, malignancies excluding NMSC, and NMSC. IRs for herpes zoster, all serious infections, and malignancies excluding NMSC remained generally stable over time. Recurrent herpes zoster was observed in 30 patients. IRs were higher (CI non-overlapping) with tofacitinib 10 mg BID versus 5 mg BID for herpes zoster and opportunistic infections excluding tuberculosis and numerically higher (CI marginally overlapping) for all serious infections; no dose-dependencies were indicated for other events.

Infections and infestations were anticipated to represent the most common class of overall AEs. IRs for serious infections were consistent between ORAL Sequel, the integrated analysis of safety data for tofacitinib [18], and pooled ORAL Sequel and Study A3921041 data [16], at 2.4, 2.7, and 2.5, respectively, as were IRs for herpes zoster between ORAL Sequel and the integrated analysis of safety data, at 3.4 and 3.9, respectively (data not reported for the pooled ORAL Sequel and Study A3921041 analysis; IR was 7.4 for herpes zoster in the Japanese population of Study A3921041 alone [15]). With respect to tuberculosis, the IR for all tofacitinib (0.2) was slightly lower compared with the IR for other opportunistic infections excluding tuberculosis (0.4) in ORAL Sequel. In the integrated analysis of safety data for tofacitinib, IRs were similar between tuberculosis (0.2) and other opportunistic infections excluding tuberculosis (0.3), and concordant with ORAL Sequel data [18]. Prior evaluation has supported the understanding that the risk of tuberculosis in patients receiving tofacitinib treatment directly varies with background tuberculosis prevalence in different geographic sub-populations [19]. ORAL Sequel IR data for tuberculosis are comparable with data for biologic DMARDs [20–22].

Of interest, the long-latency events MACE and lymphoma had IRs at 0.4 and 0.1, respectively. IRs for other AEs of interest reported in both ORAL Sequel and the integrated analysis of safety data for tofacitinib [18] were consistent: 0.8 and 0.9 for malignancies excluding NMSC, 0.7 and 0.6 for NMSC, and both 0.1 for gastrointestinal perforation (which is also comparable with tumor necrosis factor inhibitors [TNFi] [23]). Furthermore, the IRs for DVT and PE in patients receiving tofacitinib (both 0.1) were comparable to those reported in data pooled from phase 2 and phase 3 studies of tofacitinib as monotherapy or in combination with DMARDs (DVT IR of 0.1 for both tofacitinib 5 and 10 mg BID, and PE IR of 0.1 for tofacitinib 5 mg BID and 0.2 for tofacitinib 10 mg BID [24]) and in the literature for patients with RA, including those treated with DMARDs (DVT IRs of 0.45 [25] and 0.62 [26], and PE IRs of 0.26 [25] and 0.20 [26]).

In support of the consistencies noted above, safety data from LTE studies and meta-analyses of biologic and targeted synthetic DMARDs are comparable with those reported for tofacitinib. Considering discontinuation from biologic DMARDs, 62% of patients discontinued by year 10 of treatment in an LTE study of adalimumab (23% due to AEs and 12% due to lack of efficacy/disease progression) [27]; and 31% of patients discontinued by year 5 in an LTE study of subcutaneous abatacept (31% due to AEs and 7% due to lack of efficacy) [28]. Considering safety events of special interest, previous LTE studies have reported IRs for serious infections of 3.1 and 1.7 events for adalimumab (across 10 years of treatment) and subcutaneous abatacept (across 5 years of treatment), respectively [27, 28]. In the integrated analysis of safety data for baricitinib, event IRs were reported for serious infections (3.0), herpes zoster (3.3), tuberculosis (0.1), MACE (0.5), DVT (0.4), PE (0.2), malignancies excluding NMSC (0.8), lymphoma (0.08), NMSC (0.4), and gastrointestinal perforation (0.04) [29, 30]. Meta-analysis data showed that in a dataset comprising RCTs and LTE studies, the IRs for tofacitinib (serious infection 2.74; malignancies excluding NMSC 0.89) were comparable to those reported for biologic and targeted synthetic DMARDs, including abatacept, adalimumab, baricitinib, certolizumab pegol, etanercept, golimumab, infliximab, rituximab, and tocilizumab (serious infection 3.04–7.59; malignancies excluding NMSC 0.75–1.06) [31, 32], which in turn are concordant with data reported for ORAL Sequel. In addition, serious infection IRs for tofacitinib were observed to be congruous with IRs previously calculated in a 2017 meta-analysis of RCTs for biologic DMARDs, including abatacept (3.0), rituximab (3.5), tocilizumab (5.4), and TNFi (5.5), and the targeted synthetic DMARD baricitinib (4.8 for 2 mg, 3.7 for 4 mg) [31]. Taking the findings of ORAL Sequel and these data together, this supports the work of other groups concluding that the rates of serious infections for tofacitinib are within the range of those reported for biologic DMARDs (up to 12 weeks only) [33] and the targeted synthetic DMARD, baricitinib (versus placebo) [29].

AE data in ORAL Sequel indicated some differences between tofacitinib monotherapy and combination therapy. IRs for AEs leading to discontinuation in ORAL Sequel were higher (CI non-overlapping) in patients receiving tofacitinib combination therapy (7.7) compared with patients receiving tofacitinib monotherapy (5.9); SAE IRs were also numerically different (CI marginally overlapping) between patients receiving combination therapy (9.5) and tofacitinib monotherapy (8.1). In terms of AEs of special interest, IRs were higher (CI non-overlapping) in patients receiving tofacitinib as combination therapy versus monotherapy for herpes zoster. Within the subgroup of patients receiving tofacitinib as combination therapy, IRs were higher (CI non-overlapping) with tofacitinib 10 mg BID versus 5 mg BID for all serious infections, herpes zoster, and opportunistic infections excluding tuberculosis.

Laboratory variables of interest, including cholesterol, LDL, ALT, AST, and serum creatinine, remained generally stable over time*.* ALC levels gradually declined until month 48, whereafter the level stabilized. Overall, 40.6% of patients experienced moderate lymphopenia, and 30.1% of patients experienced severe lymphopenia; these events did not meet with study discontinuation criteria, per protocol. Approximately 1% of patients had a drop in lymphocyte count to < 500 cells/mm^3^ (potentially life-threatening lymphopenia); however, nearly all recovered to above 500 cells/mm^3^ upon treatment discontinuation. Additionally, five of these patients experienced a serious infection during the study; two occurred within 30 days of the patients’ lowest lymphocyte count. Although the proportion of patients who developed a serious infection within 30 days of the lowest lymphocyte count was highest among patients with potentially life-threatening lymphopenia, these results should be interpreted with caution due to the low number of patients with ALC < 500 cells/mm^3^. As per the tofacitinib prescribing information, routine clinical practice should involve evaluation of lymphocyte count at baseline, monitoring every 3 months during tofacitinib treatment, and consideration of the following actions: discontinuation if ALC reaches < 500 cells/mm^3^ (a level associated with increased risk of serious infections [18, 34]), dose reduction or interruption if ALC reaches 500–750 cells/mm^3^, and dose maintenance if ALC is ≥ 750 cells/mm^3^ [35, 36].

Clinically meaningful improvements in the signs and symptoms of RA as measured by ACR response rates, DAS28-4(ESR) improvements from baseline, and CDAI- and SDAI-defined remission rates, as well as improvements in physical functioning as measured by HAQ-DI improvements from baseline, were also maintained over time in patients who remained on tofacitinib treatment. Efficacy data for patients receiving tofacitinib monotherapy or tofacitinib plus csDMARD combination therapy were not available here, but have been previously published [17]; in this earlier analysis of data pooled from ORAL Sequel and Study A3921041, efficacy was maintained through to month 72, regardless of treatment regimen.

It is acknowledged that LTE studies are limited by enrolling eligible patients who completed preceding index studies and excluding those who developed tofacitinib treatment-related SAEs and so were discontinued from preceding index studies. As per the protocol, significant opportunistic infections or serious infection events, and certain malignancies, precluded enrollment to ORAL Sequel. LTE study populations, therefore, represent patients in whom the study drug is known to be efficacious and well tolerated, restricting full evaluation of the benefit to risk profile. It is important to note that a greater exposure (patient-years) occurred with tofacitinib 10 mg BID compared with 5 mg BID, as a result of the majority of patients enrolling from large phase 3 studies and initiating the LTE study at 10 mg BID per protocol (except for patients from China, bringing a geographic skew). Caution should be taken when interpreting results for patients with the longest tofacitinib exposure due to the relatively small patient numbers at later months. Furthermore, tofacitinib dose assignment based on using average TDD does not account for cumulative treatment exposure or dose changes over time. It also does not provide the actual dose when the event occurred; therefore, although 77% of patients remained on their study-start dose, this may have attenuated any between-dose differences for the 5 mg versus 10 mg BID tofacitinib dose. We also recognize that there may be limitations to the interpretation of efficacy results, given that all analyses were conducted “as observed” and patients not achieving an effect on treatment in the qualifying study were more likely to not enroll in the LTE study or discontinue. To this point, it should be noted that only < 4% of patients discontinued from the LTE study due to insufficient clinical response. The lack of a placebo or comparator arm also restricts benefit to risk profile evaluation. Finally, comparisons of data for combination versus monotherapy should be treated with caution because each prior qualifying index study defined the regimen for all patients within that study, and patients were not randomized to dose for the LTE study or to one regimen versus the other (monotherapy versus combination therapy). The monotherapy subgroup was also smaller (*n* = 1298) than the combination therapy subgroup (*n* = 2464).

Nevertheless, open-label LTE studies provide valuable information. A controlled setting with rigorous safety reporting, including independent adjudication of events, is facilitated. Data for drug exposure are collected over months or years, yielding a long-term safety profile. Indeed, additional long-term studies of tofacitinib are ongoing: Study A3921133 (NCT02092467), an ongoing post-marketing surveillance study, was initiated to compare the safety of tofacitinib versus TNFi in patients with RA in an open-label design. With final enrollment estimated at ~ 4400 patients, outcomes are event-driven, with malignancies excluding NMSC and the incidence of MACE being the primary outcome measures. Study A3921133 is, therefore, anticipated to provide further insights that will contribute further to the evaluation of the benefit to risk profile of tofacitinib. Furthermore, a recent publication on post-marketing surveillance experience with tofacitinib showed that no new safety risks were revealed in this real-world setting compared with the safety profile identified in the RA clinical development program [37]. As of December 2017, it is estimated that over 115,000 patients with RA worldwide have received tofacitinib (Pfizer data on file).

## Conclusions

Tofacitinib 5 mg and 10 mg BID demonstrated a consistent safety profile and sustained efficacy in this open-label LTE ORAL Sequel study of patients with RA. Safety data are reported up to 9.5 years, and efficacy data up to 8 years, based on adequate patient numbers to support conclusions. Observed IRs for SAEs, serious infections, malignancies, MACE, DVT, and PE were similar to those observed in pooled data from phase 1, 2, 3, and LTE studies, and comparable to those seen with TNFi and other biologic DMARDs. Patient-level laboratory safety data were consistent with findings from prior tofacitinib phase 2, phase 3, and LTE studies. Furthermore, the safety profile of tofacitinib in patients who initiated tofacitinib as monotherapy was generally similar to that observed when tofacitinib was initiated in combination with csDMARDs. Tofacitinib 5 mg and 10 mg BID provided sustained improvement in signs and symptoms of RA and improvements in physical function.

## Additional files

Additional file 1: **Table S1.** Summary of qualifying index studies associated with ORAL Sequel (NCT00413699).

Additional file 2: **Figure S1.** Patient disposition (a) and discontinuation over time (b). ^a^Four patients in the tofacitinib 10 mg BID arm had a missing end-of-study page. ^b^Evaluable for AEs (evaluable for laboratory data: tofacitinib 5 mg BID *n* = 1118, tofacitinib 10 mg BID *n* = 3346, and all tofacitinib *n* = 4464). ^c^Two patients in the tofacitinib 10 mg BID arm did not have recorded AEs. Safety analysis set: all patients who received at least one dose of study medication; efficacy analysis set: all patients who received at least one dose of study medication and had at least one post-baseline efficacy measurement available. Time to discontinuation: difference between the end-of-study date and first tofacitinib dose date plus 1 day; completers are censored at the end-of-study date. Study discontinuation occurred with the following scenarios: serious infections requiring antimicrobial therapy or hospitalization; opportunistic infections judged to be significant by the investigator; two sequential lymphocyte or neutrophil counts < 500 mm^3^ (neutrophil counts < 1000 mm^3^ for patients from Croatia, Czech Republic, Denmark, Germany, Ireland, Korea, Spain, Sweden, and the UK); two sequential platelet counts < 75,000 mm^3^; two sequential AST or ALT elevations > 3 times the ULN with ≥ 1 total bilirubin value > 2 times the ULN, abnormal International Normalized Ratio liver function test, or symptoms consistent with hepatic injury (or elevations > 5 times the ULN regardless); single positive HBcAb and a negative HBsAb; two sequential hemoglobins < 8.0 g/dL or a decrease > 30% from baseline; two sequential increases in serum creatinine > 100% of the average baseline/screening values (> 50% for Korea); other serious or severe AEs. Database lock: March 2, 2017. *AE* adverse event, *ALT* alanine aminotransferase, *AST* aspartate aminotransferase, *BID* twice daily, *HBcAb* hepatitis B core antibody, *HBsAb* hepatitis B surface antibody, *ULN* upper limit of normal.

Additional file 3: **Table S2.** Patient baseline demographic and disease characteristics.

Additional file 4: **Figure S2.** All-cause AEs (a) and all-cause AEs leading to discontinuation (b) over time. Baseline qualifying index study data were used for approximately 90% of patients. Database lock: March 2, 2017. *AE* adverse event, *BID* twice daily.

Additional file 5: **Table S3.** Incidence of all mortality (a) and mortality listings (b).

Additional file 6: **Figure S3.** Mean (SE) ALT (a), AST (b), hemoglobin (c), and serum creatinine (d) over time. Baseline qualifying index study data were used for approximately 90% of patients. Data for 12-month intervals are reported in the tables . Database lock: March 2, 2017. *ALT* alanine aminotransferase, *AST* aspartate aminotransferase, *BID* twice daily, *SE* standard error.

Additional file 7: **Table S4.** Confirmed AST and ALT > 1×, ≥ 2×, and ≥ 3× ULN.

Additional file 8: **Figure S4.** ACR50 (a) and ACR70 (b) response rates over time (observed). ACR calculated with respect to qualifying index study data available for approximately 90% of patients. Italicized data not reported in figure due to low patient numbers. Data for 12-month intervals are reported in the tables. Database lock: March 2, 2017. *ACR* American College of Rheumatology, *BID* twice daily.

Additional file 9: **Figure S5.** Remission as defined by CDAI (score ≤ 2.8) (a) and SDAI (score ≤ 3.3) (b) (observed). Baseline qualifying index study data were used for approximately 90% of patients. Italicized data not reported in figure due to low patient numbers. Data for 12-month intervals are reported in the tables. Database lock: March 2, 2017. *BID* twice daily, *BL* baseline, *CDAI* Clinical Disease Activity Index, *SDAI* Simplified Disease Activity Index, *SE* standard error.

## Abbreviations

ACR: American College of Rheumatology
AE: Adverse event
ALC: Absolute lymphocyte count
ALT: Alanine aminotransferase
ANC: Absolute neutrophil count
AST: Aspartate aminotransferase
BID: Twice daily
BL: Baseline
BMI: Body mass index
CDAI: Clinical Disease Activity Index
CI: Confidence interval
CRP: C-reactive protein
csDMARD: Conventional synthetic disease-modifying antirheumatic drug
DAS28-4(ESR): Disease Activity Score in 28 joints using erythrocyte sedimentation rate
DMARD: Disease-modifying antirheumatic drug
EAER: Exposure-adjusted event rate
ECG: Electrocardiogram
ESR: Erythrocyte sedimentation rate
HAQ-DI: Health Assessment Questionnaire-Disability Index
HBcAb: Hepatitis B core antibody
HBsAb: Hepatitis B surface antibody
IR: Incidence rate
LDA: Low disease activity
LDL: Low-density cholesterol
LTE: Long-term extension
MACE: Major adverse cardiovascular event
MTX: Methotrexate
NMSC: Non-melanoma skin cancer
PY: Patient-years of exposure
Q2W: Every 2 weeks
QD: Once daily
RA: Rheumatoid arthritis
RCT: Randomized controlled trial
SAE: Serious adverse event
SC: Subcutaneous
SD: Standard deviation
SDAI: Simplified Disease Activity Index
SE: Standard error
SOC: System organ class
TDD: Total daily dose
TNFi: Tumor necrosis factor inhibitor
ULN: Upper limit of normal
