# Supplementary material for: Safety and efficacy of tofacitinib for up to 9.5 years in the treatment of rheumatoid arthritis: final results of a global, open-label, long-term extension study
Source: Arthritis Res Ther. 2019 Apr 5;21:89. doi: 10.1186/s13075-019-1866-2 (PMC6451219; doi:10.1186/s13075-019-1866-2)
Supplement: Supplementary file 1 — Table S1. Summary of qualifying index studies associated with ORAL Sequel (NCT00413699). [file 13075_2019_1866_MOESM1_ESM.docx]

| **Additional file 1: Table S1** Summary of qualifying index studies associated with ORAL Sequel (NCT00413699)   \| **Clinicaltrials.gov identifier** \| **Protocol number** \| **Patients initially receiving tofacitinib, n** \| **Patient population** \| **Tofacitinib doses** \| **Control arm** \| **Study duration** \| **Patients entering Study A3921024 (NCT00413699), n** \| \| --- \| --- \| --- \| --- \| --- \| --- \| --- \| --- \| \| **Phase 1** \| \| \| \| \| \| \| \| \| NCT01262118 [1] \| A3921130 \| 36 (RA), 33 (healthy volunteers) \| Active RA and healthy volunteers \| 10 mg BID (background MTX permitted) \| None \| 6 weeks \| 26 \| \| NCT01484561 [2] \| A3921152 \| 97 \| Active RA with inadequate response to ≥ 1 DMARD \| 10 mg BID (background csDMARDs permitted) \| Placebo BID \| 6 weeks (for tofacitinib treatment) \| 119 \|  \| **Phase 2** \| \| \| \| \| \| \| \| \| --- \| --- \| --- \| --- \| --- \| --- \| --- \| --- \| \| NCT00147498 [3] \| A3921019 \| 199 \| Active RA with inadequate or unacceptable toxicity to either MTX 15 mg or greater, or to any of the following: etanercept, infliximab, or adalimumab \| 5 mg BID, 15 mg BID, 30 mg BID monotherapy \| Placebo BID \| 6 weeks \| 72 \| \| NCT00413660 [4] \| A3921025 \| 438 \| Active RA with inadequate response to MTX \| 1, 3, 5, 10, or 15 mg BID or 20 mg QD with background MTX \| Placebo \| 24 weeks \| 393 \| \| NCT00550446 [5] \| A3921035 \| 272 \| Active RA with inadequate response to ≥ 1 DMARD \| 1, 3, 5, 10, or 15 mg BID monotherapy \| Adalimumab SC 40 mg Q2W; placebo \| 24 weeks \| 296 \| \| NCT01164579 [6] \| A3921068 \| 72 \| Early active RA, MTX-naïve \| 10 mg BID plus MTX, 10 mg BID monotherapy \| MTX \| 52 weeks \| 69 \| \| NCT00976599 [7] \| A3921073 \| 15 \| Active RA with inadequate response to MTX \| 10 mg BID plus background MTX \| Placebo \| 4 weeks \| 28 \| \| NCT01059864 [8] \| A3921109 \| 111 \| Active RA \| 10 mg BID, half of patients received concomitant atorvastatin 10 mg QD for Weeks 6–12 \| None \| 12 weeks \| 92 \| \| NCT01359150 [9] \| A3921129 \| 112 \| Active RA \| 10 mg BID monotherapy (half of patients) or with background MTX \| Placebo only (half of patients) or placebo plus MTX \| 9 weeks \| 207 \| \| NCT02147587 [10] \| A3921237 \| 55 \| Moderate to severe RA with inadequate response to MTX \| 5 mg BID plus background MTX;  2–3 weeks post-herpes zoster vaccination \| Placebo \| 14 weeks \| 100 \|  \| **Phase 3** \| \| \| \| \| \| \| \| \| --- \| --- \| --- \| --- \| --- \| --- \| --- \| --- \| \| NCT00960440 [11] \| ORAL Step, A3921032 \| 399 \| Moderate to severe RA with inadequate response to TNFi \| 5 or 10 mg BID with background MTX \| Placebo (advanced to tofacitinib at Month 3) \| 6 months \| 315 \| \| NCT00847613 [12] \| ORAL Scan, A3921044 \| 797 \| Active RA with inadequate response to MTX \| 5 or 10 mg BID with background MTX \| Placebo (advanced to tofacitinib at Month 3 [non-responders] or 6 [remaining patients]) \| 24 months \| 435 \| \| NCT00814307 [13] \| ORAL Solo, A3921045 \| 610 \| Active RA with inadequate response to  ≥ 1 DMARD \| 5 or 10 mg BID monotherapy \| Placebo (advanced to tofacitinib at Month 3) \| 6 months \| 519 \| \| NCT00856544 [14] \| ORAL Sync, A3921046 \| 792 \| Active RA with inadequate response to  ≥ 1 DMARD \| 5 or 10 mg BID with background MTX \| Placebo (advanced to tofacitinib at Month 3 [non-responders] or 6 [remaining patients]) \| 12 months \| 627 \| \| NCT00853385 [15] \| ORAL Standard, A3921064 \| 513 \| Active RA with incomplete response to MTX \| 5 or 10 mg BID with background MTX \| Adalimumab 40 mg SC Q2W; placebo (patients receiving placebo were advanced to tofacitinib at Month 3 [non-responders] or 6 [remaining patients]) \| 12 months \| 525 \| \| NCT01039688 [16] \| ORAL Start, A3921069 \| 766 \| Active RA, MTX-naïve \| 5 or 10 mg BID monotherapy \| MTX \| 24 months \| 658 \| \| **Total** \| – \| 5317 \| – \| – \| – \| – \| 4481 \|   Participating countries in ORAL Sequel: Argentina, Australia, Austria, Belgium, Bosnia & Herzegovina, Brazil, Bulgaria, Canada, Chile, China, Colombia, Costa Rica, Croatia, Czech Republic, Denmark, Dominican Republic, Finland, France, Germany, Greece, Hungary, India, Ireland, Italy, Republic of Korea, Malaysia, Mexico, New Zealand, Peru, Philippines, Poland, Puerto Rico, Romania, Russia, Slovakia, Spain, Sweden, Taiwan, Thailand, Turkey, Ukraine, UK, and USA  There may be more patients per study entering ORAL Sequel compared with those initially in tofacitinib arm(s), for example, in cases where patients advanced to tofacitinib from placebo if eligible  *BID* twice daily, *csDMARD* conventional synthetic disease-modifying antirheumatic drug, *DMARD* disease-modifying antirheumatic drug, *MTX* methotrexate, *Q2W* every 2 weeks, *QD* once daily, *RA* rheumatoid arthritis, *SC* subcutaneous, *TNFi* tumor necrosis factor inhibitor |
| --- | --- | --- | --- | --- | --- | --- | --- | --- | --- | --- | --- | --- | --- | --- | --- | --- | --- | --- | --- | --- | --- | --- | --- | --- | --- | --- | --- | --- | --- | --- | --- | --- | --- | --- | --- | --- | --- | --- | --- | --- | --- | --- | --- | --- | --- | --- | --- | --- | --- | --- | --- | --- | --- | --- | --- | --- | --- | --- | --- | --- | --- | --- | --- | --- | --- | --- | --- | --- | --- | --- | --- | --- | --- | --- | --- | --- | --- | --- | --- | --- | --- | --- | --- | --- | --- | --- | --- | --- | --- | --- | --- | --- | --- | --- | --- | --- | --- | --- | --- | --- | --- | --- | --- | --- | --- | --- | --- | --- | --- | --- | --- | --- | --- | --- | --- | --- | --- | --- | --- | --- | --- | --- | --- | --- | --- | --- | --- | --- | --- | --- | --- | --- | --- | --- | --- | --- | --- | --- | --- | --- | --- | --- | --- | --- | --- | --- | --- | --- | --- | --- | --- | --- | --- | --- | --- | --- | --- | --- | --- | --- | --- | --- | --- | --- | --- | --- | --- | --- |

**References**

1. Charles-Schoeman C, Fleischmann R, Davignon J, Schwartz H, Turner SM, Beysen C, et al. Potential mechanisms leading to the abnormal lipid profile in patients with rheumatoid arthritis versus healthy volunteers and reversal by tofacitinib. Arthritis Rheumatol. 2015;67:616-25.

2. Kremer JM, Kivitz AJ, Simon-Campos JA, Nasonov EL, Tony HP, Lee SK, et al. Evaluation of the effect of tofacitinib on measured glomerular filtration rate in patients with active rheumatoid arthritis: results from a randomised controlled trial. Arthritis Res Ther. 2015;17:95.

3. Kremer JM, Bloom BJ, Breedveld FC, Coombs JH, Fletcher MP, Gruben D, et al. The safety and efficacy of a JAK inhibitor in patients with active rheumatoid arthritis: results of a double-blind, placebo-controlled phase IIa trial of three dosage levels of CP-690,550 versus placebo. Arthritis Rheum. 2009;60:1895-905.

4. Kremer JM, Cohen S, Wilkinson BE, Connell CA, French JL, Gomez-Reino J, et al. A phase IIb dose-ranging study of the oral JAK inhibitor tofacitinib (CP-690,550) versus placebo in combination with background methotrexate in patients with active rheumatoid arthritis and an inadequate response to methotrexate alone. Arthritis Rheum. 2012;64:970-81.

5. Fleischmann R, Cutolo M, Genovese MC, Lee EB, Kanik KS, Sadis S, et al. Phase IIb dose-ranging study of the oral JAK inhibitor tofacitinib (CP-690,550) or adalimumab monotherapy versus placebo in patients with active rheumatoid arthritis with an inadequate response to disease-modifying antirheumatic drugs. Arthritis Rheum. 2012;64:617-29.

6. Conaghan PG, Østergaard M, Bowes MA, Wu C, Fuerst T, van der Heijde D, et al. Comparing the effects of tofacitinib, methotrexate and the combination, on bone marrow oedema, synovitis and bone erosion in methotrexate-naive, early active rheumatoid arthritis: results of an exploratory randomised MRI study incorporating semiquantitative and quantitative techniques. Ann Rheum Dis. 2016;75:1024-33.

7. Boyle DL, Soma K, Hodge J, Kavanaugh A, Mandel D, Mease P, et al. The JAK inhibitor tofacitinib suppresses synovial JAK1-STAT signalling in rheumatoid arthritis. Ann Rheum Dis. 2015;74:1311-6.

8. McInnes IB, Kim HY, Lee SH, Mandel D, Song YW, Connell CA, et al. Open-label tofacitinib and double-blind atorvastatin in rheumatoid arthritis patients: a randomised study. Ann Rheum Dis. 2014;73:124-31.

9. Winthrop KL, Silverfield J, Racewicz A, Neal J, Lee EB, Hrycaj P, et al. The effect of tofacitinib on pneumococcal and influenza vaccine responses in rheumatoid arthritis. Ann Rheum Dis. 2016;75:687-95.

10. Winthrop KL, Wouters AG, Choy EH, Soma K, Hodge JA, Nduaka CI, et al. The safety and immunogenicity of live zoster vaccination in patients with rheumatoid arthritis before starting tofacitinib: a randomized Phase II trial. Arthritis Rheumatol. 2017;69:1969-77.

11. Burmester GR, Blanco R, Charles-Schoeman C, Wollenhaupt J, Zerbini C, Benda B, et al. Tofacitinib (CP-690,550) in combination with methotrexate in patients with active rheumatoid arthritis with an inadequate response to tumour necrosis factor inhibitors: a randomised phase 3 trial. Lancet. 2013;381:451-60.

12. van der Heijde D, Tanaka Y, Fleischmann R, Keystone E, Kremer J, Zerbini C, et al. Tofacitinib (CP-690,550) in patients with rheumatoid arthritis receiving methotrexate: twelve-month data from a twenty-four-month phase III randomized radiographic study. Arthritis Rheum. 2013;65:559-70.

13. Fleischmann R, Kremer J, Cush J, Schulze-Koops H, Connell CA, Bradley JD, et al. Placebo-controlled trial of tofacitinib monotherapy in rheumatoid arthritis. N Engl J Med. 2012;367:495-507.

14. Kremer J, Li Z-G, Hall S, Fleischmann R, Genovese M, Martin-Mola E, et al. Tofacitinib in combination with nonbiologic disease-modifying antirheumatic drugs in patients with active rheumatoid arthritis: a randomized trial. Ann Intern Med. 2013;159:253-61.

15. van Vollenhoven RF, Fleischmann R, Cohen S, Lee EB, García Meijide JA, Wagner S, et al. Tofacitinib or adalimumab versus placebo in rheumatoid arthritis. N Engl J Med. 2012;367:508-19.

16. Lee EB, Fleischmann R, Hall S, Wilkinson B, Bradley J, Gruben D, et al. Tofacitinib versus methotrexate in rheumatoid arthritis. N Engl J Med. 2014;370:2377-86.
