# Supplementary figures and images for: Safety and efficacy of tofacitinib for up to 9.5 years in the treatment of rheumatoid arthritis: final results of a global, open-label, long-term extension study
Source: Arthritis Res Ther. 2019 Apr 5;21:89. doi: 10.1186/s13075-019-1866-2 (PMC6451219; doi:10.1186/s13075-019-1866-2)

**a**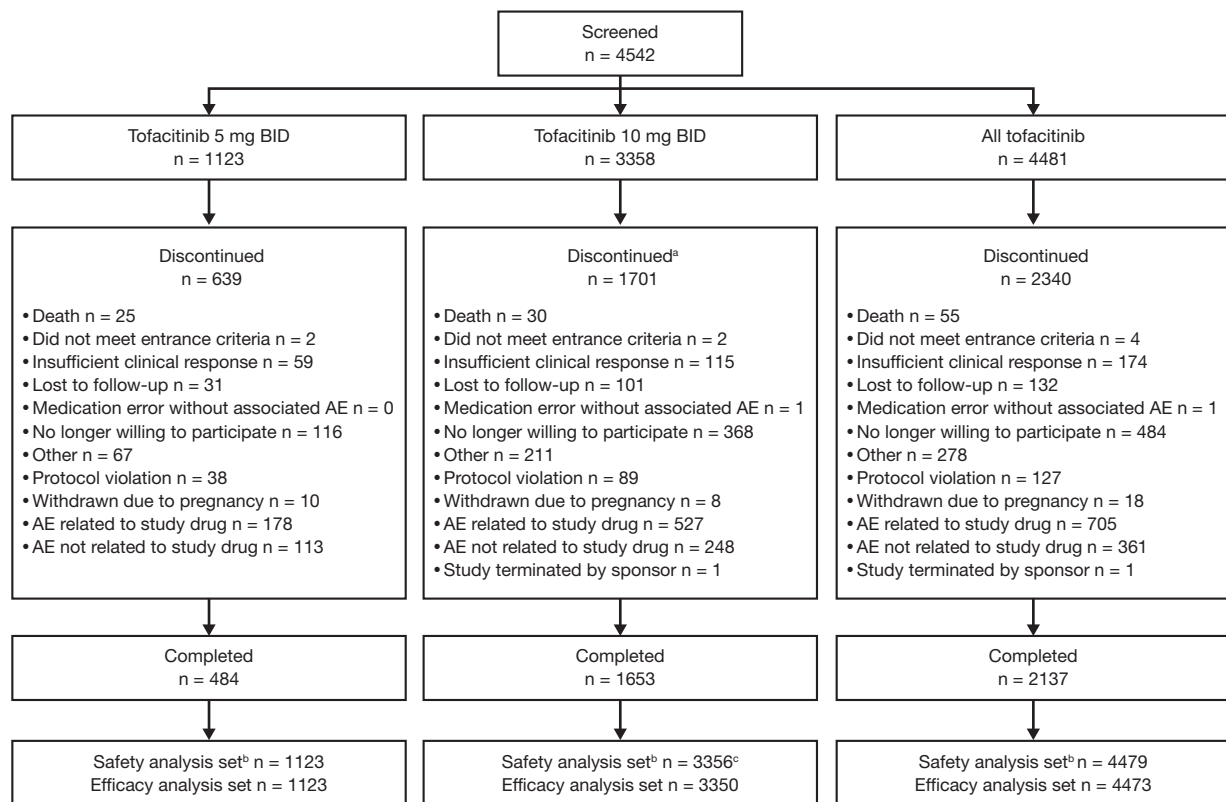**b**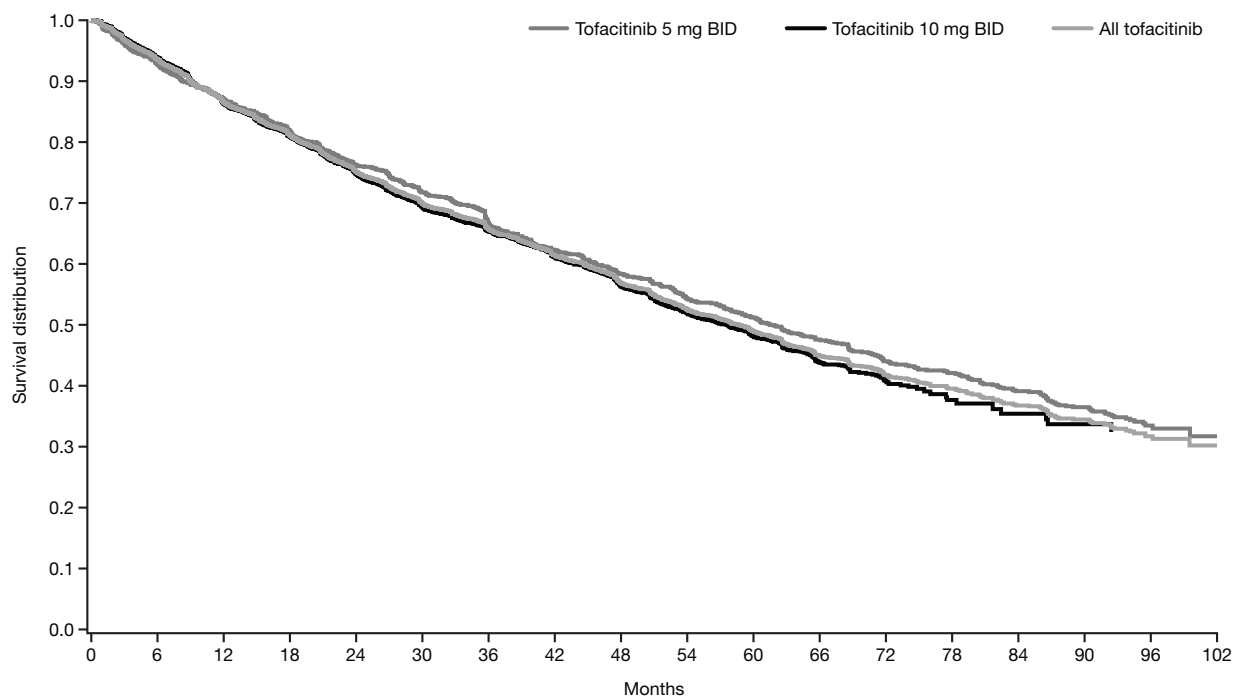

Supplement: Supplementary file 2 — Figure S1. Patient disposition (a) and discontinuation over time (b). aFour patients in the tofacitinib 10 mg BID arm had a missing end-of-study page. bEvaluable for AEs (evaluable for laboratory data: tofacitinib 5 mg BID n = 1118, tofacitinib 10 mg BID n = 3346, and all tofacitinib n = 4464). cTwo patients in the tofacitinib 10 mg BID arm did not have recorded AEs. Safety analysis set: all patients who received at least one dose of study medication; efficacy analysis set: all patients who received at least one dose of study medication and had at least one post-baseline efficacy measurement available. Time to discontinuation: difference between the end-of-study date and first tofacitinib dose date plus 1 day; completers are censored at the end-of-study date. Study discontinuation occurred with the following scenarios: serious infections requiring antimicrobial therapy or hospitalization; opportunistic infections judged to be significant by the investigator; two sequential lymphocyte or neutrophil counts < 500 mm3 (neutrophil counts < 1000 mm3 for patients from Croatia, Czech Republic, Denmark, Germany, Ireland, Korea, Spain, Sweden, and the UK); two sequential platelet counts < 75,000 mm3; two sequential AST or ALT elevations > 3 times the ULN with ≥ 1 total bilirubin value > 2 times the ULN, abnormal International Normalized Ratio liver function test, or symptoms consistent with hepatic injury (or elevations > 5 times the ULN regardless); single positive HBcAb and a negative HBsAb; two sequential hemoglobins < 8.0 g/dL or a decrease > 30% from baseline; two sequential increases in serum creatinine > 100% of the average baseline/screening values (> 50% for Korea); other serious or severe AEs. Database lock: March 2, 2017. AE adverse event, ALT alanine aminotransferase, AST aspartate aminotransferase, BID twice daily, HBcAb hepatitis B core antibody, HBsAb hepatitis B surface antibody, ULN upper limit of normal. [file 13075_2019_1866_MOESM2_ESM.pdf]

**a**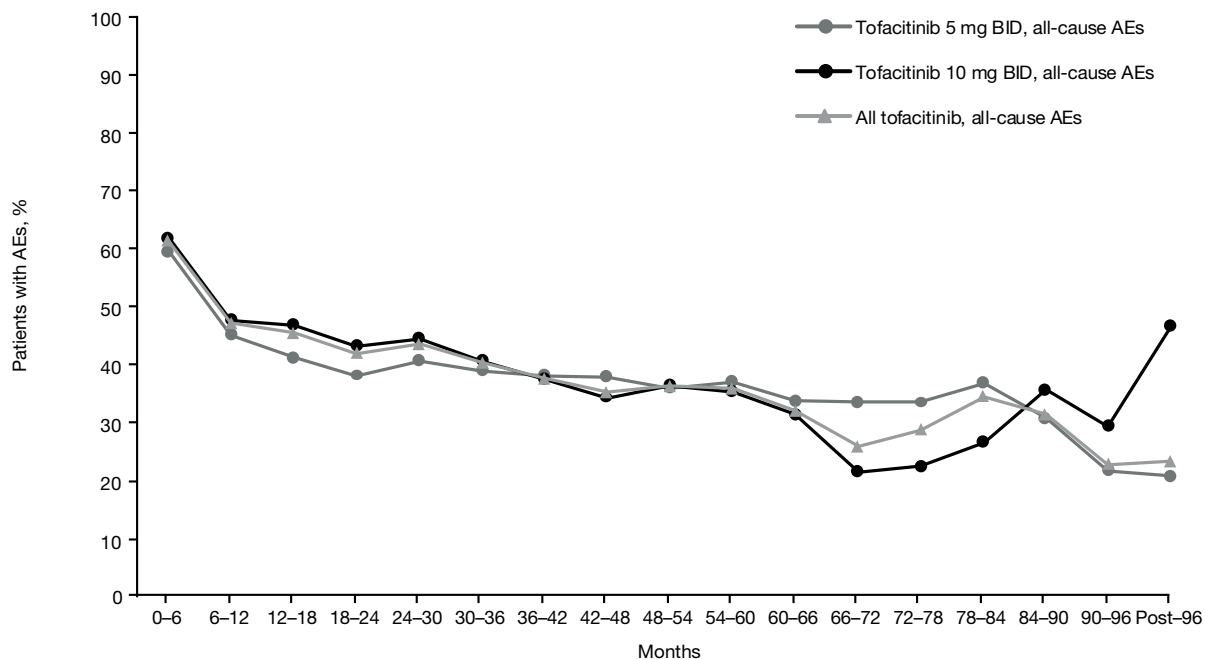**b**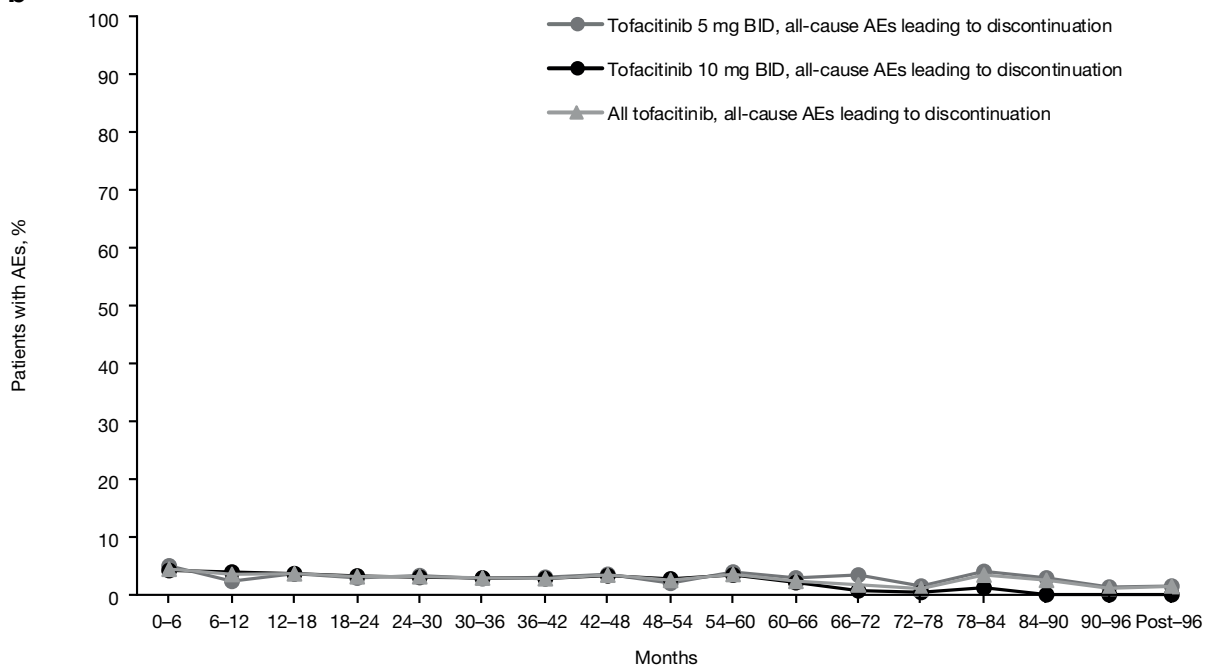

Supplement: Supplementary file 4 — Figure S2. All-cause AEs (a) and all-cause AEs leading to discontinuation (b) over time. Baseline qualifying index study data were used for approximately 90% of patients. Database lock: March 2, 2017. AE adverse event, BID twice daily. [file 13075_2019_1866_MOESM4_ESM.pdf]

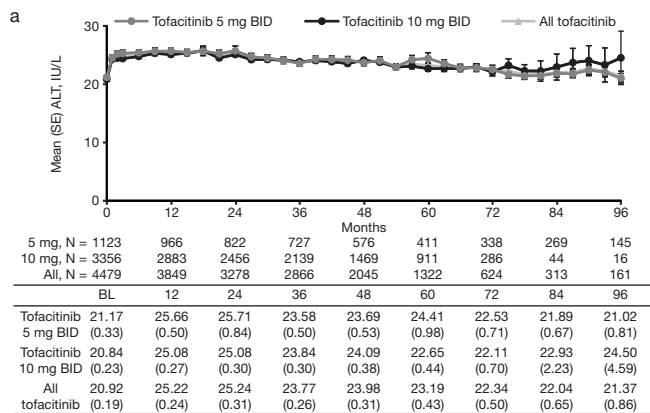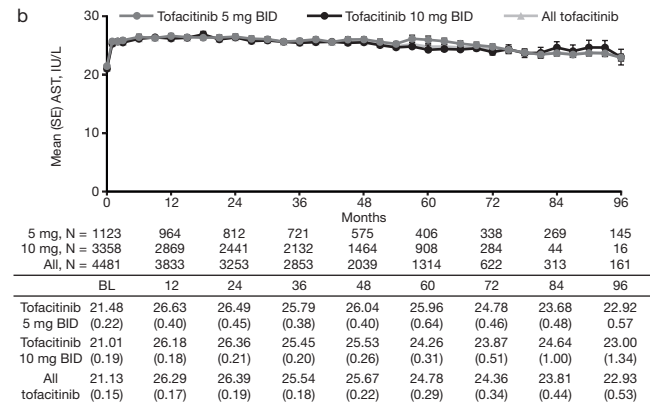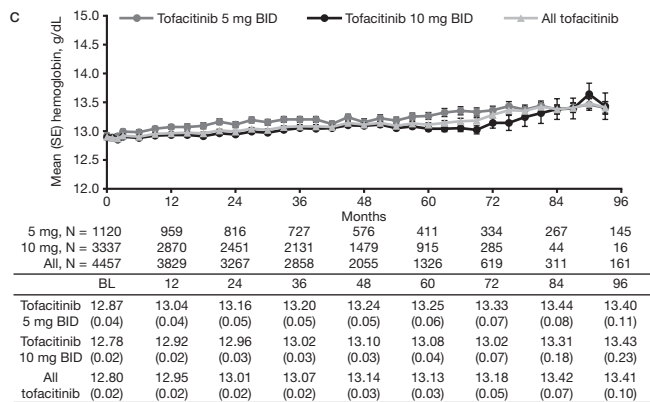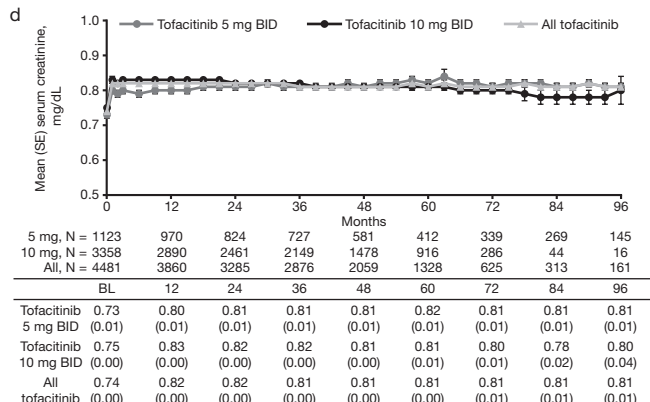

Supplement: Supplementary file 6 — Figure S3. Mean (SE) ALT (a), AST (b), hemoglobin (c), and serum creatinine (d) over time. Baseline qualifying index study data were used for approximately 90% of patients. Data for 12-month intervals are reported in the tables . Database lock: March 2, 2017. ALT alanine aminotransferase, AST aspartate aminotransferase, BID twice daily, SE standard error. [file 13075_2019_1866_MOESM6_ESM.pdf]

**a**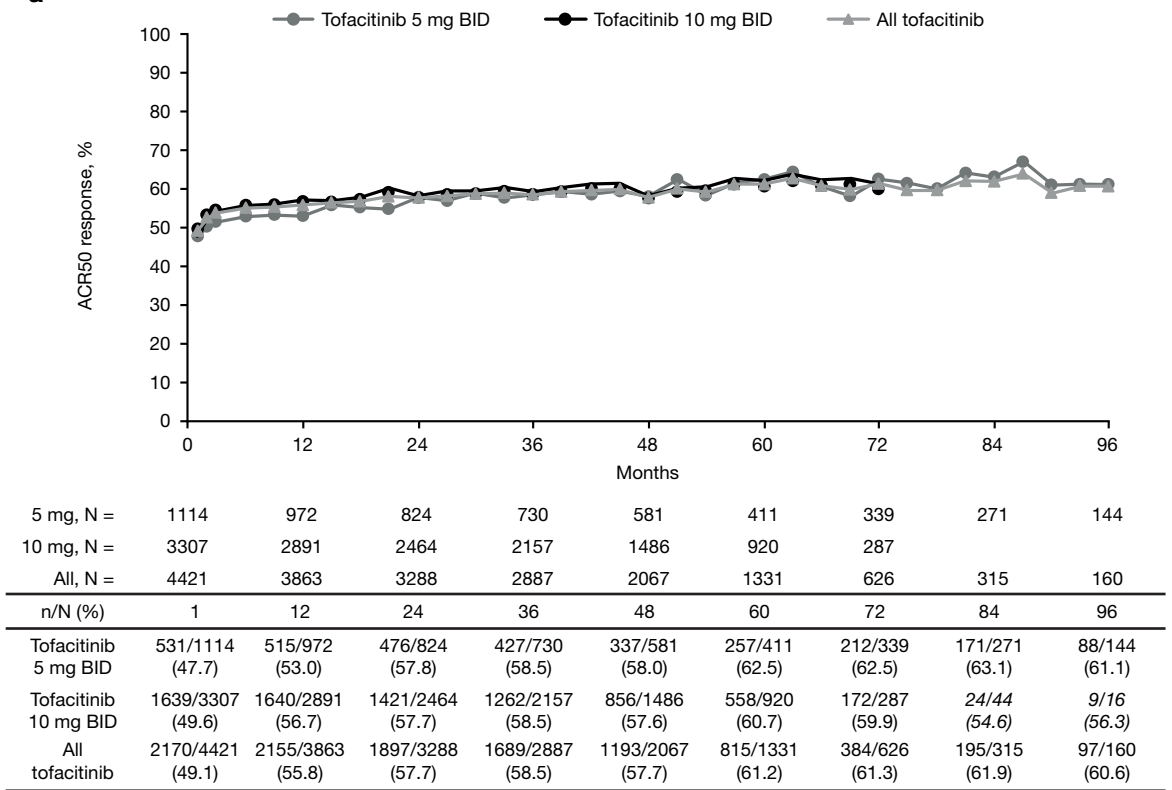**b**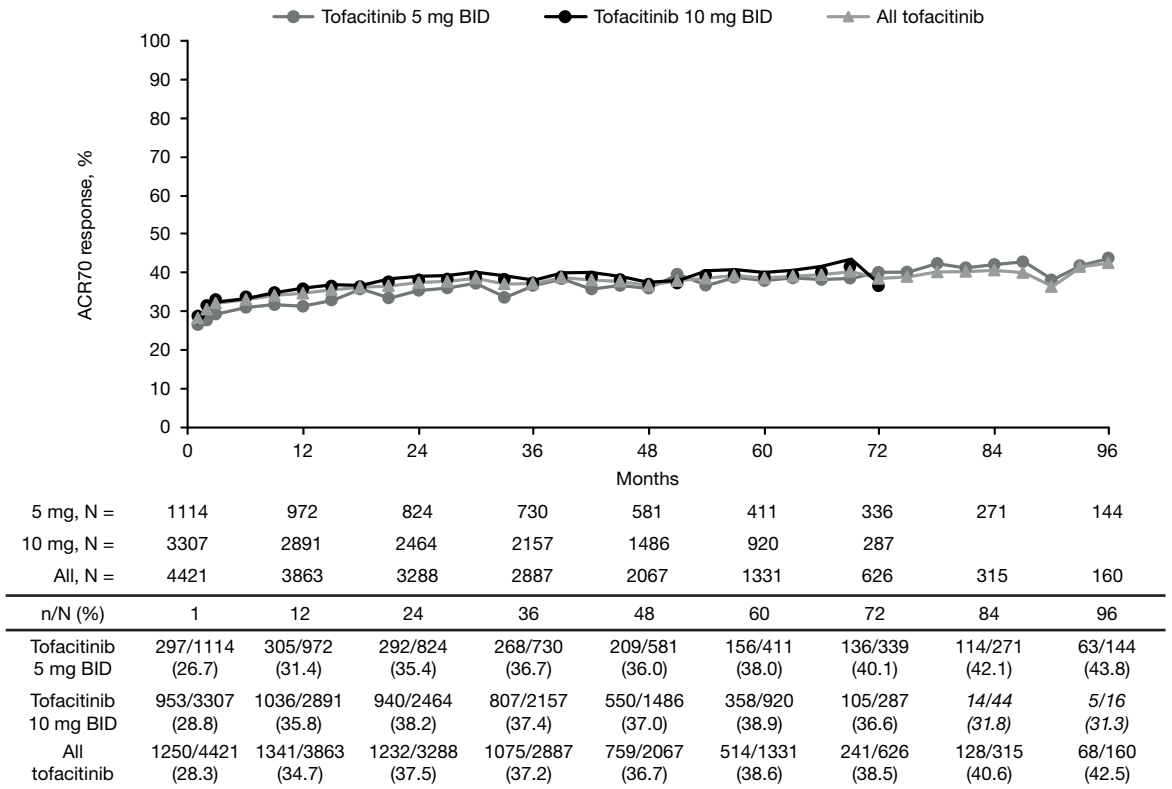

Supplement: Supplementary file 8 — Figure S4. ACR50 (a) and ACR70 (b) response rates over time (observed). ACR calculated with respect to qualifying index study data available for approximately 90% of patients. Italicized data not reported in figure due to low patient numbers. Data for 12-month intervals are reported in the tables. Database lock: March 2, 2017. ACR American College of Rheumatology, BID twice daily. [file 13075_2019_1866_MOESM8_ESM.pdf]

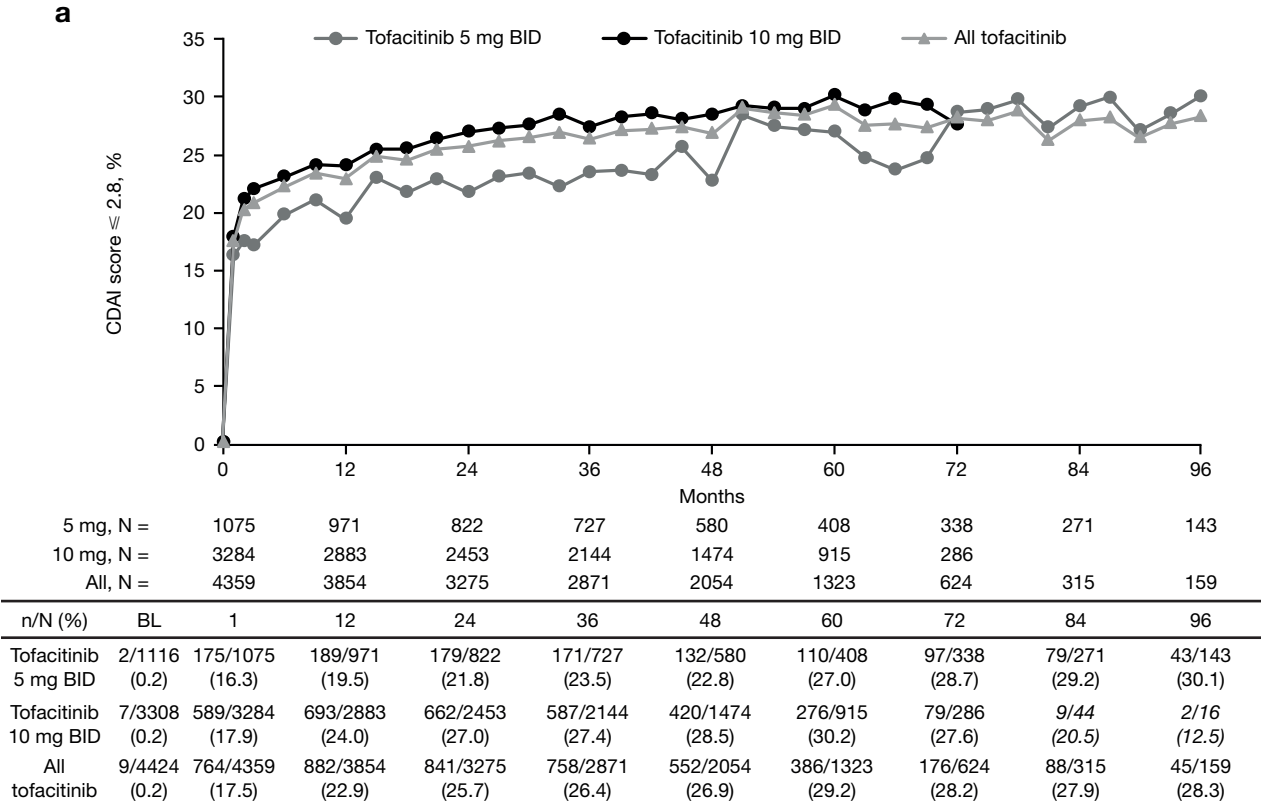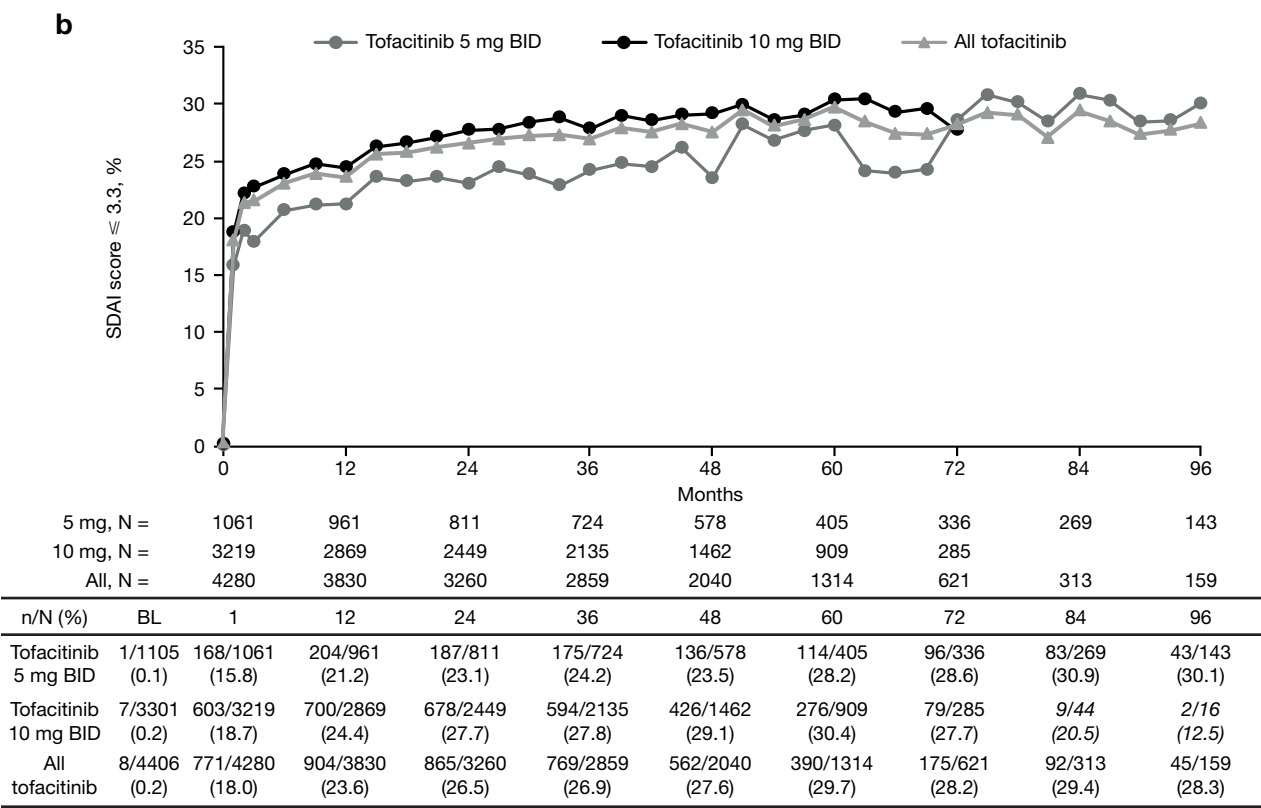

Supplement: Supplementary file 9 — Figure S5. Remission as defined by CDAI (score ≤ 2.8) (a) and SDAI (score ≤ 3.3) (b) (observed). Baseline qualifying index study data were used for approximately 90% of patients. Italicized data not reported in figure due to low patient numbers. Data for 12-month intervals are reported in the tables. Database lock: March 2, 2017. BID twice daily, BL baseline, CDAI Clinical Disease Activity Index, SDAI Simplified Disease Activity Index, SE standard error. [file 13075_2019_1866_MOESM9_ESM.pdf]
