# Supplementary material for: Safety and efficacy of tofacitinib for up to 9.5 years in the treatment of rheumatoid arthritis: final results of a global, open-label, long-term extension study
Source: Arthritis Res Ther. 2019 Apr 5;21:89. doi: 10.1186/s13075-019-1866-2 (PMC6451219; doi:10.1186/s13075-019-1866-2)
Supplement: Supplementary file 3 — Table S2. Patient baseline demographic and disease characteristics. [file 13075_2019_1866_MOESM3_ESM.docx]

| **Additional file 3: Table S2** Patient baseline demographic and disease characteristics   \|  \| **Tofacitinib 5 mg BID**  **(n = 1123)** \| **Tofacitinib 10 mg BID**  **(n = 3358)** \| **All tofacitinib**  **(n = 4481)** \| \| --- \| --- \| --- \| --- \| \| Age, mean (SD) \| 54.0 (12.1) \| 53.0 (11.5) \| 53.3 (11.6) \| \| Female, n (%) \| 927 (82.5) \| 2744 (81.7) \| 3671 (81.9) \| \| Race, n (%)  White  Black  Asian  Hispanic  Other \| 745 (66.3)  28 (2.5)  225 (20.0)  14 (1.2)  111 (9.9) \| 2430 (72.4)  112 (3.3)  475 (14.1)  0  341 (10.2) \| 3175 (70.9)  140 (3.1)  700 (15.6)  14 (0.3)  452 (10.1) \| \| BMI, mean (SD) kg/m^2^ \| 27.0 (5.9) \| 27.7 (6.5) \| 27.5 (6.4) \| \| Smoking status, n (%)  Never-smoker  Smoker  Ex-smoker  Unknown \| 702 (62.5)  177 (15.8)  173 (15.4)  71 (6.3) \| 2136 (63.6)  596 (17.7)  622 (18.5)  4 (0.1) \| 2838 (63.3)  773 (17.3)  795 (17.7)  75 (1.7) \| \| Tender joint count, mean (SD) \| 22.8 (13.5) \| 25.7 (14.9) \| 25.0 (14.6) \| \| Swollen joint count, mean (SD) \| 14.7 (8.8) \| 15.4 (9.3) \| 15.2 (9.2) \| \| ESR, mean (SD) \| 46.2 (25.4) \| 49.1 (27.1) \| 48.5 (26.7) \| \| CRP, mean (SD) \| 18.3 (22.6) \| 18.0 (23.2) \| 18.1 (23.1) \| \| DAS28-4(ESR), mean (SD) \| 6.3 (1.0) \| 6.4 (1.0) \| 6.4 (1.0) \| \| Duration of disease, mean (SD) years \| 8.6 (8.4) \| 7.7 (7.9) \| 7.9 (8.0) \| \| CDAI, mean (SD) \| 36.4 (13.0) \| 37.2 (13.1) \| 37.0 (13.1) \| \| SDAI, mean (SD) \| 38.3 (13.7) \| 39.0 (13.8) \| 38.8 (13.7) \| \| Positive for rheumatoid factor, n (%) \| 791 (75.3) \| 2245 (72.2) \| 3036 (73.0) \| \| Positive for anti-citrullinated protein antibody, n (%) \| 329 (78.9) \| 2297 (75.9) \| 2626 (76.3) \| \| Herpes zoster, n (%)^a^  Had chicken pox  Received chicken pox vaccine  Received herpes zoster vaccine  Ever had shingles (herpes zoster) once during lifetime \| 243 (21.6)  19 (1.7)  44 (3.9)  87 (7.8) \| 1048 (31.2)  134 (4.0)  72 (2.1)  366 (10.9) \| 1291 (28.8)  153 (3.4)  116 (2.6)  453 (10.1) \|   ^a^Many patients unknown for one or more options due to incomplete data collection for duration of study  Baseline qualifying index study data were used for approximately 90% of patients (index baseline data applicable to efficacy/laboratory variables, with disease duration calculated at index baseline; LTE baseline data applicable to demographic variables)  Database lock: March 2, 2017  *BID* twice daily, *BMI* body mass index, *CDAI* Clinical Disease Activity Index, *CRP* C-reactive protein, *DAS28-4(ESR)* Disease Activity Score in 28 joints using erythrocyte sedimentation rate, *ESR* erythrocyte sedimentation rate, *LTE* long-term extension, *SD* standard deviation, *SDAI* Simplified Disease Activity Index |
| --- | --- | --- | --- | --- | --- | --- | --- | --- | --- | --- | --- | --- | --- | --- | --- | --- | --- | --- | --- | --- | --- | --- | --- | --- | --- | --- | --- | --- | --- | --- | --- | --- | --- | --- | --- | --- | --- | --- | --- | --- | --- | --- | --- | --- | --- | --- | --- | --- | --- | --- | --- | --- | --- | --- | --- | --- | --- | --- | --- | --- | --- | --- | --- | --- | --- | --- | --- | --- |
