# Supplementary material for: Safety and efficacy of tofacitinib for up to 9.5 years in the treatment of rheumatoid arthritis: final results of a global, open-label, long-term extension study
Source: Arthritis Res Ther. 2019 Apr 5;21:89. doi: 10.1186/s13075-019-1866-2 (PMC6451219; doi:10.1186/s13075-019-1866-2)
Supplement: Supplementary file 5 — Table S3. Incidence of all mortality (a) and mortality listings (b). [file 13075_2019_1866_MOESM5_ESM.docx]

**Additional file 5**: Table S3 Incidence of all mortality (**a**) and mortality listings (**b**)

**a**

|  | **All patients** | | | **Patients receiving tofacitinib as combination therapy  (stay-on background csDMARDs)** | | | **Patients receiving tofacitinib as monotherapy  (stay-on monotherapy)** | | |  |
| --- | --- | --- | --- | --- | --- | --- | --- | --- | --- | --- |
|  | **Tofacitinib 5 mg BID** | **Tofacitinib 10 mg BID** | **All tofacitinib** | **Tofacitinib 5 mg BID** | **Tofacitinib 10 mg BID** | **All tofacitinib** | **Tofacitinib 5 mg BID** | **Tofacitinib 10 mg BID** | **All tofacitinib** |  |
| **IR (95% CI) [n/N]** | | | | | | | | | | |
| Mortality^a^ | 0.4  (0.2, 0.6) [19/1123] | 0.2 (0.1, 0.3)  [25/3358] | 0.3  (0.2, 0.4)  [44/4481] | 0.4  (0.2, 0.7)  [9/630] | 0.2  (0.1, 0.4)  [14/1834] | 0.3  (0.2, 0.4)  [23/2464] | 0.8  (0.4, 1.4)  [10/305] | 0.2  (0.1, 0.5)  [8/993] | 0.4  (0.2, 0.6)  [18/1298] |  |

**^a^**All mortality (including that attributed to a cardiovascular event, infections, malignancy, or other)

IR is the number of patients with events per 100 patient-years

Database lock: March 2, 2017

*BID* twice daily, *CI* confidence interval, *csDMARD* conventional synthetic disease-modifying antirheumatic drug, *IR* incidence rate

**b**

| **Gender** | **Age** | **Race** | **Country** | **Study day** | **AE by system  organ class** | **AE by preferred term** | **Causality** |
| --- | --- | --- | --- | --- | --- | --- | --- |
| **All patients, tofacitinib 5 mg BID (N=19)** | | | | | | | |
| Female | 67 | Other | Mexico | 812 | Respiratory, thoracic and mediastinal disorders | Chronic obstructive pulmonary disease / respiratory failure | Unrelated |
| Male | 44 | White | Brazil | 833 | Infections and infestations | Appendicitis | Related |
| Male | 45 | White | United States | 1354 | Cardiac disorders | Arrhythmia | Unrelated |
| Male | 49 | White | United States | 2656 | Cardiac disorders | Cardiac arrest | Unrelated |
| Female | 62 | White | Argentina | 1313 | Infections and infestations | Pneumonia | Related |
| Female | 71 | White | Chile | 878 | Nervous system disorders | Cerebrovascular accident | Unrelated |
| Male | 51 | White | United States | 2418 | Cardiac disorders | Myocardial infarction | Unrelated |
| Female | 73 | White | Czech Republic | 1600 | Cardiac disorders | Cardiopulmonary failure | Unrelated |
| Male | 68 | White | Czech Republic | 986 | Nervous system disorders | Cerebral haemorrhage | Unrelated |
| Female | 59 | White | Greece | 2600 | Nervous system disorders | Ruptured cerebral aneurysm | Unrelated |
| Female | 63 | White | Bulgaria | 2098 | General disorders and administration site conditions | Death | Related |
| Female | 51 | White | Germany | 1908 | General disorders and administration site conditions | Multiple organ dysfunction syndrome / pneumonic sepsis | Related |
| Female | 64 | White | Slovakia | 1830 | Hepatobiliary disorders | Cholelithiasis / pancreatitis acute | Unrelated |
| Male | 58 | White | Slovakia | 2084 | Renal and urinary disorders | Acute kidney injury | Unrelated |
| Female | 70 | White | Chile | 980 | Neoplasms benign, malignant and unspecified (incl. cysts and polyps) | Gallbladder cancer | Unrelated |
| Female | 56 | White | United States | 558 | Cardiac disorders | Cardio-respiratory arrest | Unrelated |
| Female | 61 | White | Chile | 718 | Cardiac disorders | Cardiogenic shock | Related |
| Male | 66 | White | Czech Republic | 207 | Respiratory, thoracic and mediastinal disorders | Acute respiratory distress syndrome / septic shock | Related |
| Female | 63 | White | Ukraine | 1289 | Infections and infestations | Encephalitis | Related |
| **All patients, tofacitinib 10 mg BID (N=25)** | | | | | | | |
| Male | 52 | White | United States | 2016 | Neoplasms benign, malignant and unspecified (incl. cysts and polyps) | Lung cancer metastatic | Related |
| Male | 56 | White | United States | 416 | Cardiac disorders | Arrhythmia | Unrelated |
| Female | 70 | Other | Argentina | 200 | General disorders and administration site conditions | Death | Related |
| Female | 60 | White | Argentina | 668 | Cardiac disorders | Cardio-respiratory arrest | Unrelated |
| Female | 70 | White | Austria | 1769 | Cardiac disorders | Myocardial infarction | Unrelated |
| Female | 47 | Asian | Taiwan | 1373 | Nervous system disorders | Brain stem infarction | Unrelated |
| Female | 49 | White | United States | 1010 | Neoplasms benign, malignant and unspecified (incl. cysts and polyps) | Lung cancer metastatic | Related |
| Male | 66 | White | United States | 1841 | Neoplasms benign, malignant and unspecified (incl. cysts and polyps) | Lung neoplasm malignant | Related |
| Female | 60 | White | United States | 1268 | Injury, poisoning and procedural complications | Road traffic accident | Unrelated |
| Male | 47 | Other | Brazil | 2041 | Respiratory, thoracic and mediastinal disorders | Interstitial lung disease / septic shock | Unrelated |
| Female | 59 | Other | Mexico | 1344 | Nervous system disorders | Hemorrhagic stroke | Related |
| Male | 76 | White | Russian Federation | 2020 | Infections and infestations | Pneumonia necrotizing | Related |
| Female | 61 | White | Finland | 1919 | General disorders and administration site conditions | Sudden death | Related |
| Female | 63 | White | United States | 2225 | Cardiac disorders | Cardio-respiratory arrest | Unrelated |
| Female | 46 | Asian | China | 1160 | Infections and infestations | Encephalitis | Related |
| Female | 73 | Asian | Thailand | 1224 | Infections and infestations | Pneumonia bacterial | Related |
| Male | 34 | White | Czech Republic | 1 | Psychiatric disorders | Completed suicide | Unrelated |
| Female | 38 | Other | Mexico | 1435 | Nervous system disorders | Cerebral hemorrhage | Related |
| Female | 63 | White | Croatia | 1798 | Respiratory, thoracic and mediastinal disorders | Pulmonary embolism | Unrelated |
| Male | 54 | White | Bulgaria | 1558 | Infections and infestations | Pneumonia | Related |
| Female | 71 | White | United States | 1131 | Nervous system disorders | Cerebral hemorrhage | Unrelated |
| Female | 72 | White | United States | 326 | Infections and infestations | Clostridium difficile colitis | Related |
| Female | 43 | White | United States | 1399 | Vascular disorders | Arteriosclerosis | Unrelated |
| Female | 61 | White | Russian Federation | 794 | Nervous system disorders | Cerebral infarction | Unrelated |
| Male | 64 | White | United States | 82 | Cardiac disorders | Cardiac arrest | Unrelated |

*AE* adverse event, *BID* twice daily
