# Supplementary material for: Safety and efficacy of tofacitinib for up to 9.5 years in the treatment of rheumatoid arthritis: final results of a global, open-label, long-term extension study
Source: Arthritis Res Ther. 2019 Apr 5;21:89. doi: 10.1186/s13075-019-1866-2 (PMC6451219; doi:10.1186/s13075-019-1866-2)
Supplement: Supplementary file 7 — Table S4. Confirmed AST and ALT > 1×, ≥ 2×, and ≥ 3× ULN. [file 13075_2019_1866_MOESM7_ESM.docx]

| **Additional file 7: Table S4** Confirmed AST and ALT > 1x, ≥ 2x, and ≥ 3x ULN   \|  \| \| **All patients** \| \| \| **Patients receiving tofacitinib as combination therapy  (stay-on background csDMARDs)** \| \| \| **Patients receiving tofacitinib as monotherapy  (stay-on monotherapy)** \| \| \| \| --- \| --- \| --- \| --- \| --- \| --- \| --- \| --- \| --- \| --- \| --- \| \|  \| \| **Tofacitinib  5 mg BID**  **(N = 1102)** \| **Tofacitinib 10 mg BID**  **(N = 3308)** \| **All tofacitinib**  **(N = 4410)** \| **Tofacitinib 5 mg BID**  **(N = 616)** \| **Tofacitinib 10 mg BID**  **(N = 1801)** \| **All tofacitinib**  **(N = 2417)** \| **Tofacitinib 5 mg BID**  **(N = 298)** \| **Tofacitinib 10 mg BID**  **(N = 976)** \| **All tofacitinib**  **(N = 1274)** \| \| **ALT, n (%)** \| > 1x ULN \| 305 (27.7) \| 887 (26.8) \| 1192 (27.0) \| 152 (24.7) \| 488 (27.1) \| 640 (26.5) \| 81 (27.2) \| 233 (23.9) \| 314 (24.6) \| \| ≥ 2x ULN \| 76 (6.9) \| 182 (5.5) \| 258 (5.9) \| 34 (5.5) \| 83 (4.6) \| 117 (4.8) \| 19 (6.4) \| 50 (5.1) \| 69 (5.4) \| \| ≥ 3x ULN \| 26 (2.4) \| 69 (2.1) \| 95 (2.2) \| 13 (2.1) \| 36 (2.0) \| 49 (2.0) \| 7 (2.3) \| 16 (1.6) \| 23 (1.8) \| \| **AST, n (%)** \| > 1x ULN \| 277 (25.1) \| 758 (22.9) \| 1035 (23.5) \| 147 (23.9) \| 402 (22.3) \| 549 (22.7) \| 70 (23.5) \| 200 (20.5) \| 270 (21.2) \| \| ≥ 2x ULN \| 42 (3.8) \| 94 (2.8) \| 136 (3.1) \| 16 (2.6) \| 47 (2.6) \| 63 (2.6) \| 14 (4.7) \| 28 (2.9) \| 42 (3.3) \| \| ≥ 3x ULN \| 14 (1.3) \| 35 (1.1) \| 49 (1.1) \| 8 (1.3) \| 22 (1.2) \| 30 (1.2) \| 3 (1.0) \| 9 (0.9) \| 12 (0.9) \|   Database lock: March 2, 2017 *ALT* alanine aminotransferase, *AST* aspartate aminotransferase, *BID* twice daily, *csDMARD* conventional synthetic disease-modifying antirheumatic drug, *ULN* upper limit of normal |
| --- | --- | --- | --- | --- | --- | --- | --- | --- | --- | --- | --- | --- | --- | --- | --- | --- | --- | --- | --- | --- | --- | --- | --- | --- | --- | --- | --- | --- | --- | --- | --- | --- | --- | --- | --- | --- | --- | --- | --- | --- | --- | --- | --- | --- | --- | --- | --- | --- | --- | --- | --- | --- | --- | --- | --- | --- | --- | --- | --- | --- | --- | --- | --- | --- | --- | --- | --- | --- | --- | --- | --- | --- | --- | --- | --- | --- | --- | --- | --- | --- | --- | --- | --- | --- |
